# Professional Grief in Cancer Care—A Scoping Review

**DOI:** 10.1002/pon.70156

**Published:** 2025-04-25

**Authors:** Svenja Wandke, Hannah Führes, Mareike Rutenkröger, Klaus Lang, Martin Härter, Karin Oechsle, Isabelle Scholl

**Affiliations:** ^1^ Department of Medical Psychology Center for Psychosocial Medicine University Medical Center Hamburg‐Eppendorf Hamburg Germany; ^2^ II. Department of Medicine University Medical Center Hamburg‐Eppendorf Hamburg Germany; ^3^ Psychotherapeutic Practice Munich Germany; ^4^ Palliative Care Unit Department of Oncology, Hematology and BMT University Medical Center Hamburg‐Eppendorf Hamburg Germany

**Keywords:** cancer, grief over patient loss, healthcare professionals, oncology, professional grief, scoping review

## Abstract

**Objective:**

Healthcare professionals (HCPs) in cancer care often face patient deaths, yet there is a notable absence of comprehensive evidence regarding their grief. This scoping review seeks to identify key aspects of professional grief in cancer care and give an overview pertaining its' conceptualization and frequency and intensity.

**Methods:**

The primary search covered three databases (MEDLINE, PSYNDEX, and PsycINFO). Two independent reviewers assessed 2248 records, selecting 34 eligible articles.

**Results:**

Most studies originated from North America and Israel, with limited evidence from the global south, East Asia and Europe, as well as few quantitative studies. HCPs exhibit classic grief symptoms (such as sadness) and distinct features (e.g., feelings of guilt) in response to patient deaths, though a clear definition and measures of professional grief are lacking. Grief frequency varies highly (from 23% to 100%).

**Conclusions:**

Future research should refine definitions and measures to better support HCPs in effectively managing professional grief in cancer care.

## 1 Background

When individuals experience the loss of family members or friends, the typical and anticipated reaction is grief, which is the human, emotional response to the loss of someone or something significant [1, 2, 3]. However, evidence suggests that this notion of significance also applies to professional relationships, such as those between healthcare professionals (HCPs) and their patients. In cancer care, HCPs often form close bonds with patients due to the extensive and sometimes long‐term care they provide [4, 5]. As cancer remains a leading global cause of death [3, 6], the loss of patients is inherent to HCPs working in this field. The distress HCPs might experience after a patient's death is sometimes referred to as “professional grief,” a phenomenon that hasn't been comprehensively evaluated [7]. Emerging evidence suggests that exposure to patient suffering and death can lead to compassion fatigue, a state of emotional exhaustion and diminished empathy resulting from prolonged caregiving [8, 9]. Compassion fatigue comprises secondary traumatic stress (STS), which arises from exposure to others' trauma, and burnout, which is linked to work‐related stressors such as excessive workload and lack of support [3, 8, 10]. The relationship between patient loss, professional grief, and compassion fatigue remains complex and insufficiently understood [3, 11, 12]. It is unclear whether professional grief precedes compassion fatigue and burnout, or whether it is a co‐occurring phenomenon. Some evidence suggests that how HCPs experience and cope with patient deaths—that is, their professional grief—may influence their risk of developing burnout and STS [3, 8, 12].

Recognizing professional grief as a critical factor in this process is essential for improving HCP well‐being and mitigating the negative consequences of burnout and compassion fatigue. In high‐mortality fields such as oncology, understanding the role of professional grief could inform interventions aimed at fostering resilience and sustaining compassionate care [3, 11, 12, 13, 14]. Thus, this scoping review aimed to consolidate knowledge on professional grief in cancer care by providing a comprehensive synthesis of existing evidence.

## 2 Methods

### 2.1 Protocol and Registration

To conduct this scoping review, we applied the Arksey & O'Malley framework and based our study protocol on the PRISMA‐ScR‐Checklist [15, 16]. We initially registered our study protocol on September 15, 2022 with the open science framework (https://doi.org/10.17605/OSF.IO/R7B8H), and then updated it on March 13, 2023 (https://doi.org/10.17605/OSF.IO/Y5QHN), to refine our inclusion criteria.

### 2.2 Eligibility Criteria

We included studies focusing on HCPs' responses to patient death, coping strategies, and related aspects in cancer care, regardless of whether they used the term “professional grief.” Original research using qualitative, quantitative, or mixed‐methods, as well as systematic reviews, were included. Articles had to be in English, German, or French (see Supporting Information S1 for the checklist of inclusion criteria). We excluded articles on non‐patient‐related grief, non‐cancer contexts, and studies involving HCPs still in training.

### 2.3 Information Sources and Screening Process

We conducted primary searches between June and September 2022, updating in April 2024 in MEDLINE, PsycINFO, and PSYNDEX databases. Thus, databases were searched from their inception to April 2024. The search strategy for MEDLINE is detailed in Supporting Information S2. Records were screened in Rayyan, with duplicates removed [17]. Reference tracking was also performed. Pre‐screening was conducted to establish inclusion criteria consistency. Titles and abstracts were independently screened by two reviewers, with disagreements resolved through discussion. Full‐text assessment was done by two reviewers independently (S.W., H.F.), with disagreements resolved through discussion with a third reviewer (M.R.).

### 2.4 Data Charting Process

The first author developed a data charting form, which was iteratively updated and independently charted by a second reviewer (L.W., cf. acknowledgments), and checked by a third reviewer (M.R.), who recoded results when necessary. Disagreements were resolved through discussion. The charting included study characteristics, aims, key findings, terminology used, professional grief characteristics, and its association with related constructs. Upon charting, all eligible studies were categorized inductively based on their aim and key findings. Categories were developed by the first author and agreed upon by all authors.

## 3 Results

### 3.1 Included Studies

**FIGURE 1:**
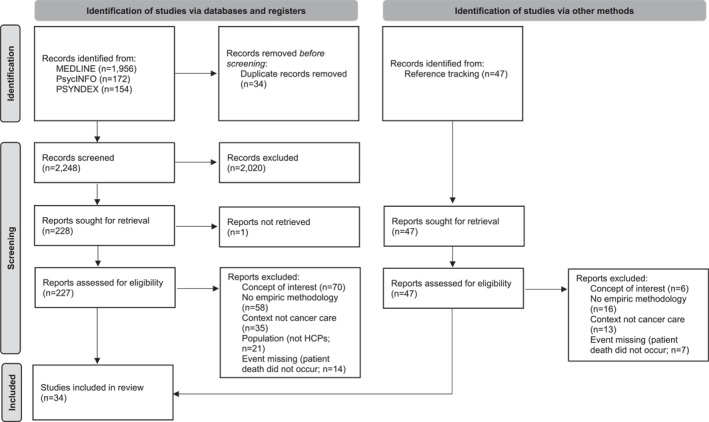
Study selection.

After removing duplicates, we found 2248 records in electronic databases and 47 through reference tracking. We screened 274 full‐text reports, resulting in 34 eligible studies (see Figure 1). These studies, published between 1994 and 2023, mainly originated in Canada (*n* = 10), the United States (*n* = 9), and Israel (*n* = 7). Most studies were qualitative, with only a fifth using validated quantitative measures to assess professional grief. They primarily focused on physicians (*n* = 16) and nurses (*n* = 11). Characteristics of these studies are detailed in Table 1.

**TABLE 1:** Study characteristics.

Author (year), journal	Country	Methodology	Study design	Study population (*n*, group of HCPs)	Nomenclature for PG	Aim & key findings
The experience of professional grief (*n* = 10)						
Chau et al. (2009), *Archives of Internal Medicine* [18]	Canada	Quantitative (online survey)	Cross‐sectional	*n* = 535, medical and radiation oncologists and palliative care physicians	—	Aim: Exploration of physician's participation in bereavement practices Palliative care physicians have a tendency to be more involved in bereavement care and feel less (no) guilt after a patient's death 64% at least sometimes took part in bereavement rituals; barriers to bereavement follow‐up: Lack of time or resources
Conte (2014), *Journal of Hospice & Palliative Nursing* [19]	USA	Qualitative (unstructured interviews)	Cross‐sectional	*n* = 11, pediatric oncology nurses	Experiences with work‐related loss, work‐related grief	Aim: Report pediatric oncology nurses' experiences with work‐related loss and grief Characteristics of professional grief: Feelings of guilt and helplessness, expected suppression of emotions/staying professional (“keeping it together”); professional grief identified as a form of grief (“It's not the same as losing a family member, but it is still a loss.”) Having a close relationship with patients and their families was two‐fold, it provided a source of meaning to nurses, but also intensified feelings of loss/grief, guilt and helplessness. Nurses also identified positive impacts of their exposure to chronic patient death, For example a special appreciation for life
Feldstein & Buschman (1995), *Cancer Nursing* [20]	USA	Quantitative (paper pencil questionnaire)^c^	Cross‐sectional	*n* = 50, oncology nurses	Grief, chronic compounded grief), professional grief	Aim: Determine whether nurses who leave the field of oncology experience more grief than those who stay All nurses exhibited grief, but those who stayed in the field and those who changed field did not differ significantly in levels or sources of grief or available support
Granek et al. (2012), *Archives of Internal Medicine* [8]	Canada	Qualitative (semi‐structured interviews)	Cross‐sectional	*n* = 20, oncologists	Grief over patient loss, oncologists' grief	Aim: Exploration of oncologists grief’ over deceased patients and effects on personal and professional lives 30% of sample reported crying and feelings of guilt (signs of professional grief) Patient loss is a unique affective experience, distinct from grief in everyday life (smoke‐like quality); influences oncologists, their affect and personal lives as well as other patients; dysfunctional coping with patient loss might not only affect oncologists themselves negatively but also their living patients due to for example withdrawal or alteration of treatment decisions
Granek et al. (2012), *Journal of Hospice & Palliative Nursing* [11]	Canada	Qualitative (semi‐structured interviews)	Cross‐sectional	*n* = 20, oncologists	Grief over patient loss, oncologists' grief, physician's grief	Aim: Explore what intensifies oncologist's grief over patient deaths Oncologists found patient deaths particularly distressing due to either relational factors, such as long‐term treatment or close relationships, or contextual factors, such as unrealistic expectations from the patient's family (Workplace) culture influenced how oncologists acknowledged and expressed grief
Granek et al. (2015), *Blood Cancer* [21]	Canada	Qualitative (semi‐structured interviews)	Cross‐sectional	*n* = 21, pediatric oncologists	Grief, reactions to patient death	Aim: Investigate pediatric oncologists' reactions to patient deaths Reactions to patient deaths included sadness, crying, sleep deprivation, feelings of guilt, helplessness and exhaustion, being drained, feeling physically ill, a sense of personal loss, self‐questioning and a sense of failure while still maintaining a professional role (“keeping it together”); guilt stemmed either from causing pain while delivering bad news and feeling responsible for them or “survivors guilt” (moving on with one's own life after patient deaths); guilt in pediatric oncologists was elevated in comparison to oncologists dealing with adult populations Patient deaths impacted oncologists both negatively and positively, in their professional as well as their personal life Duration of grief is inconsistent, from lasting a few moments or hours to even months/years; even when acute grief subsides some patient deaths had a life‐long impact on oncologists Chronic patient deaths and anticipatory grief were identified as especially burdensome, as well as being part of the families grieving experience additionally to one's own grief
Granek et al. (2017), *Journal of Oncology Practice* [22]	Israel	Mixed methods (semi‐structured interviews & survey)	Cross‐sectional	Qualitative *n* = 22; quantitative *n* = 79, oncologists	Grief, grief over patient death, feelings of grief, physician grief	Aim: Explore the impact of frequent patient deaths on oncologists lives Frequent patient death held positive and negative consequences for oncologists personal and professional lives; including personality changes (becoming more negative) while also being better able to keep in mind what is important in life (79%), as well as feeling exhausted (62%) and burned out (76%) while still learning from patients and improving care (67%) Chronic exposure to patient death seemed to normalize death and dying for most oncologists (53%), while some reported more death anxiety (24%)
Granek et al. (2017), *Psycho‐Oncology* [23]	Israel	Qualitative (semi‐structured interviews)	Cross‐sectional	*n* = 22, oncologists	Grief, grief symptoms over patient death, patient‐related grief	Aim: Explore oncologist's grief symptoms over patient death and to identify why and which losses are particularly challenging Grief symptoms: Behavioral (e.g., crying, trouble with sleep), cognitive (e.g., rumination, self‐doubt), physical (chest/general discomfort, fatigue), and emotional symptoms (sadness, anxiety, helplessness, guilt, irritability, loss, relief) Difficult patient losses due to: unexpected deaths, patient's young age, identification and/or close relationships with patients, death with suffering, unrealistic expectations/physician blame/unprepared families, close relationship with the family and strong impact of a patient's disease on his/her children (e.g., cancer due to genetic origins)
Kaplan (2000), *Omega* [24]	USA	Qualitative (structured interviews)	Cross‐sectional	*n* = 15, pediatric nurses	Grief, bereavement, grief‐like reactions, disenfranchised grief	Aim: Explore emotional experiences of pediatric nurses in palliative care, formulate a model of caregiver grief 10 out of 15 participants expressed grief (67%) Participants experienced a form of “emotional tension”/grief, including feelings of sadness, loneliness, emptiness and anger as well as joy and relief, which shares elements with the grief over a loved one but also is a distinct phenomenon Distinct elements of professional grief: Grieving process takes less time and is not (as) overwhelming since no “internalized loved object” was lost due to the less intimate and always temporary relationship between HCPs and patients Due to the inexistence of a institutional support system, time constraints and societal pressures nurses were unable to express grief (disenfranchised grief), which is troublesome since finding ways to cope with grief is was identified as crucial to stay within this field of work
Kaur et al. (2021), *Indian Journal of Palliative Care* [25]	India	Qualitative (semi‐structured interviews)	Cross‐sectional	*n* = 15, mixed sample (7 nurses; 6 physicians; 1 counselor; 1 social worker)	Grief	Aim: Explore needs of cancer palliative care professionals in India All participants reported grief (100%) “Death and grief” as one of five emerging themes: All participants experienced challenges within the context of death, dying and grief, especially when deceased patients were young or suffering Reactions to patient deaths included feelings of sadness, helplessness, but also relief, crying or apathy
Influences on and of professional grief (*n* = 4)						
Engler‐Gross et al. (2020), *Psycho‐Oncology* [2]	Israel	Quantitative (survey) ^a^	Cross‐sectional	*n* = 60, psycho‐oncologists	Grief	Aim: Evaluate how grief reactions influence the development of compassion fatigue in psycho‐oncologists and how this relation is moderated by social acknowledgment Sample showed moderate to high levels of grief (*M* = 27.83, SD = 7.50) Grief showed positive correlations with secondary traumatic stress and burnout and negative associations with social acknowledgment Participants showing high levels of grief and low social acknowledgment had significantly higher secondary traumatic stress scores than those who expressed either medium levels of grief and high social acknowledgment or low levels of grief and medium social acknowledgment
Hayuni et al. (2019), *Psycho‐Oncology* [26]	Israel	Quantitative (survey) ^a^	Cross‐sectional	*n* = 71, oncologists	Grief	Aim: Examine the role of compassion fatigue as a mediator between empathy and grief Sample showed moderate to high levels of grief (*M* = 26.21, SD = 8.84), which correlated positively and significantly with secondary traumatic stress, burnout and personal distress; perspective taking and grief had a significant, negative association Mediation model: Secondary traumatic stress and burnout fully accounted for the relationship between perspective taking and personal distress (components of empathy) and grief
Laor‐Mayaany et al. (2020), *Supportive Care in Cancer* [3]	Israel	Quantitative (survey) ^a^	Cross‐sectional	*n* = 74, oncologists	Grief	Aim: Investigate the impact of secondary traumatic stress, sense of failure and grief on compassion fatigue in oncologists Participants showed moderate to high levels of grief (*M* = 26.21, SD = 8.96) Grief showed significant, positive correlations with secondary traumatic stress and burnout, grief and sense of failure combined even predicted secondary traumatic stress und burnout significantly
Shi et al. (2022), *Psycho‐Oncology* [5]	China	Quantitative (survey) ^a^	Cross‐sectional	*n* = 794, oncology nurses	Grief	Aim: Examine oncology nurses‘ grief, show it's mediating effect between empathy and compassion fatigue Sample showed moderate levels of grief (*M* = 26.06, SD = 10.81) Grief and perspective taking was negatively associated while personal distress, secondary traumatic stress and burnout were positively associated The relationship between empathy and secondary traumatic stress was partially mediated by grief
Coping with patient death (*n* = 5)						
Corn et al. (2010), *Oncologist* [4]	Israel	Quantitative (online survey)	Cross‐sectional	*n* = 126, oncologists	—	Aim: Define the frequency with which oncologists take part in bereavement practices and barriers to participation 29% of oncologists participated in face‐to‐face bereavement, 45% in indirect practices and 26% did not participate in any bereavement rituals All bereavement practices increased significantly when physicians felt like they had a “special bond” with the deceased patient while being spiritual but not religious influenced the participation in bereavement rituals positively for all patients combined Most frequent barriers to participation in bereavement rituals: Time constraints (65%), fear of burnout (61%) and the felt need to maintain boundaries (55%)
Granek et al. (2013), *Death Studies* [27]	Canada	Qualitative (semi‐structured interviews)	Cross‐sectional	*n* = 20, oncologists	Grief, grief over patient loss	Aim: Existence of institutional protocols on how to handle patient death and identification of oncologists' coping strategies when faced with patient loss Explicit hospital guidelines on what to do when a patient dies, do not exist, even though patient death is a common event in oncology; oncologists did not formulate individual protocols either Oncologists attended bereavement rituals, met or spoke with the bereaved family and sent a condolence card Other coping strategies: Seeking social support, coping with activities (e.g., taking vacations), faith, compartmentalization/denial or withdrawing from patients or their families
Granek et al. (2016), *Supportive Care in Cancer* [7]	Israel	Qualitative (semi‐structured interviews)	Cross‐sectional	*n* = 22, oncologists	Grief, oncologists' grief, grief over patient death	Aim: Exploration of oncologists' coping with patient death in Israel Strategies facilitating successful coping with patient death: Cognitive (e.g., accepting death), behavioral (hobbies, vacations etc.), relational (e.g., social support), professional (work‐life‐boundaries, withdrawing from patients, etc.), and spiritual (e.g., faith) Barriers to successful coping: Male gender and related stereotypes (“men are strong”), difficulties accessing social support (e.g., due to stigma around “seeking help”) and difficulties creating emotional boundaries
Granek et al. (2016), *Journal of Psychosocial Oncology* [28]	Canada	Qualitative (semi‐structured interviews)	Cross‐sectional	*n* = 21, pediatric oncologists	Grief, grief over patient death	Aim: Qualitative examination of pediatric oncologists' coping strategies Coping framework: Patient death may be coped with via engagement and disengagement coping Engagement coping includes primary (emotion regulation such as social support and problem solving such as supporting families at the end of life) and secondary control responses (cognitive restructuring such as conducting research or distraction such as hobbies or vacations) Disengagement coping is less desirable since it leads to voluntary avoidance; this may include withdrawing from patients and families at the end of life
Lee et al. (2023), *Cancer Nursing* [29]	South Korea	Qualitaive (non‐structured interviews)	Cross‐sectional study	*n* = 10, oncology nurses	Psychological burden of a patient's death	Aim: Exploration of oncology nurses' coping strategies after patient deaths Positive coping strategies included: Reflection (e.g., thinking about on's own mortality), readjusting one's own values (e.g., prioritizing family), improving as a medical care provider, participating in religious activities and talking to friends and/or family members Negative coping strategies included: Avoiding the patient/the end‐of‐life‐situation, either behaviorally (e.g., not going to see the patient as often) or psychologically (e.g., withdrawing emotionally), drinking alcohol and binge eating
Institutional support and interventions (*n* = 6)						
Granek et al. (2012), *Supportive Care in Cancer* [30]	Canada	Qualitative (semi‐structured interviews)	Cross‐sectional	*n* = 20, oncologists	Grief, grief over patient loss	Aim: Investigate oncologist's needs for institutional support when faced with patient death Four types of institutional support were identified as helpful: (1) training, information and education, for example fact sheets (2) acknowledgment and validation of grief, for example specific forums to share grief experiences (3) institutional psychosocial support, for example grief counselors available to staff and (4) vacations and sabbaticals
Hinds et al. (1994), *Journal of Pediatric Nursing* [31]	USA	Quantitative (survey)^c^	Interventional study, pre‐post‐design	*n* = 59, pediatric oncology nurses	Grief, bereavement	Aim: Evaluation of grief workshop on grief symptoms in pediatric oncology nurses Oncology nurses showed mostly moderate grief scores, lower than reference groups grieving the loss of a family member; somatization scores were elevated in comparison to the reference group Grief and perceived stress seem to be moderately to strongly associated, however the workshop itself did not affect levels of grief
Keene et al. (2010), *Journal of Pediatric Nursing* [32]	USA	Quantitative (survey)	Cross‐sectional	*n* = 676, mixed sample (374 nurses, 101 physicians, 55 child life specialists, 37 social workers, 109 other)	Grief, bereavement, professional distress	Aim: Evaluate the impact of bereavement debriefing sessions on grief 113 grief debriefing sessions were held for the 494 deaths from 2002 to 2005 (23%), requested mostly by oncology services Most frequently cited reasons for a request were professional distress (97 of 113 sessions) and sudden or unexpected patient death (39 of 113 sessions); having a long‐term relationship with a dying patient or their family was identified as especially troublesome Most participants found the intervention helpful, informative and meaningful; more frequent participation in sessions seemed to improve self‐management of grief and (multidisciplinary) collaboration
Macpherson (2008), *Journal of Pediatric Oncology Nursing* [33]	USA	Mixed methods (survey and audio recordings)	Interventional study, pre‐post‐design	*n* = 6, pediatric oncology nurses	Grief, loss overload	Aim: Evaluation of peer‐supported story‐telling and meaning‐making on grief Even though participants struggled to find the time to do the sessions, they expressed positive feelings about the intervention and felt social support Comments evaluating the intervention's impact on grief were conflicting; the sessions seemed more impactful in reducing grief when participants had experienced special patient deaths (*r* = 0.93, *p* ≤ 0.01)
Rettig et al. (2020), *Journal of Holistic Nursing* [34]	USA	Qualitative	Observational study	—	Grief, grief over patient loss	Aim: Evaluation of the implementation of an “remembrance & renewal” program (R&R; installing a specific space to “relieve stress, reflect on the lives of patients, and remember them in a meaningful way” for example through lighting a candle or experiencing a guided relaxation) R&R spaces were frequently used by HCPs and described as helpful in taking some time to reflect and practice self‐care; visits doubled from 2017 to 2018 and the trend seemed to continue
Rettig et al. (2023), *Journal of Holistic Nursing* [35]	USA	Quantitative (survey)	Cross‐sectional	*n* = 105, mixed sample of HCPs working in a comprehensive cancer hospital	Grief, healthcare worker grief	Aim: Examine HCPs knowledge of and engagement with the “remembrance & renewal” (R&R) program 77% of participants knew about R&R, 59% participated in the program; of those who participated once, all planned to attend future events No physicians participated in the survey, therefore data is only available concerning nurses and other employee groups Time and patient needs were the most frequent barriers to participation, while increasing age, length of time in the profession and years of education were indicators of participation Engagement with the program was mostly associated with calmness and reflection
The experience of professional grief AND coping with patient death (*n* = 7)						
Chew et al. (2021), *Journal of Advanced Nursing* [36]	Singapore	Qualitative (semi‐structured interviews)	Cross‐sectional	*n* = 12, pediatric nurses	Grief	Aim: Describe new oncology nurses' experiences, challenges and coping strategies with the death of pediatric patients Nurses reported a negative emotional impact of a pediatric patient deaths (including sadness, guilt and fear), feeling disoriented and anxious while trying to remain “professional” as well as feelings of guilt and failure after a patient's death, feeling like assumptions about this job and reality did not align Coping with pediatric patient death included seeking support from colleagues (e.g., retelling good memories together was helpful), relying on one's own spiritual beliefs and reframing each patient death as an opportunity to learn how to cope Some nurses expressed the wish for a “sharing platform” for example a focus group to help nurses, especially new ones, to cope with patient deaths
Granek et al. (2017), *Current Oncology* [37]	Canada	Quantitative (survey)	Cross‐sectional	*n* = 98, oncologists	Grief	Aim: Identify what makes patient death more emotionally difficult for oncologists and how oncologists cope with patient death Factors mainly contributing to difficult patient loss were: patient age, long‐term management of a patient, unexpected disease outcomes Oncologists used diverse coping strategies, the most prevalent one was seeking peer support
Granek et al. (2017), *Supportive Care in Cancer* [38]	Canada & Israel	Quantitative (survey)	Cross‐sectional	*n* = 177, oncologists	Grief, grief over patient death	Aim: Examine relationship between negative attitudes towards expressing emotion following patient death and burnout in oncologists and explore oncologists' preferences for interventions Negative attitudes towards expressing emotion following patient death and burnout were significantly correlated (*r* = 0.26, *p* < 0.01) Oncologist's preferences in coping with patient death varied, ranging from receiving emotional support (e.g., validation of grief over patient loss as part of working in oncology; 86%) and training on how to cope with patient death as part of for example fellowship training (80%) or seminars in general (63%) to having vacations (83%) or even doing research (58%)
Puente‐Fernández et al. (2020), *Journal of Nursing Scholarship* [39]	Spain	Systematic review of qualitative research	—	*n* = 17 studies	Emotional distress, clinicians‘ grief, grief‐related emotions	Aim: Conduct a review to summarize the attitude of nurses towards death. Patient death seems to have a large negative emotional impact on nurses, which they lack training in dealing with; successful coping with patient death is influenced by (a) the nurses‘ emotional connection with the patient and their family, (b) the families‘ attitude towards death, and (c) the nurses‘ training and experience with patient death
Treggalles & Lowrie (2018), *Australian Occupational Therapy Journal* [40]	Australia	Qualitative (semi‐structured interviews)	Cross‐sectional	*n* = 6, occupational therapists	Professional grief	Aim: Occupational therapists' experience and coping strategies to deal with professional grief in palliative care settings Self‐knowledge and especially being aware of one's own emotional “triggers” as well as keeping “mindful” and healthy boundaries were identified as essential Having an authentic emotional connection to their patients was seen incremental to feel a sense of value and satisfaction within the professional role, despite causing emotional distress in the occurrence of a patient's death; having time and space to reflect on one's feelings (e.g., holidays) and finding closure was essential to manage these emotional connections Participants identified a range of benefits of working in palliative care, such as “appreciation of time” or “what is important in life”
Zambrano et al. (2014), *Palliative & Support Care* [41]	Australia	Qualitative (semi‐structured interviews)	Cross‐sectional	*n* = 7, palliative care physicians	Grief at a professional level	Aims: Assembling palliative care physician's experiences when dealing with patient death and their coping mechanism Palliative care physicians described their role within the healthcare professions as special Emotional impact of a patient's death may include feelings of sadness, sometimes guilt but also relief; professional grief was seen as distinct from grief after personal losses (e.g., more frequent, but also more short lived) Overall physicians found their work to be rewarding and worth the costs of for example being constantly reminded of their own mortality and feeling isolated Coping strategies were either problem‐ (e.g., maintaining a work‐life‐balance) or emotion‐focused (e.g., setting boundaries), additionally physicians used meaning‐making and seeking support from team members as coping strategies
The experience of professional grief AND institutional support and interventions (*n* = 1)						
Gomes Maia da Sena et al. (2023), *Psicooncología* [42]	Brazil	Qualitative (semi‐structured interviews)	Cross‐sectional	*n* = 23, mixed sample, working in pediatric oncology	Grief	Aim: Analyzing HCPs experiences with grief within the context of palliative care in pediatric oncology HCPs experience (anticipatory) grief due to patient deterioration and loss of relationships, often unrecognized due to societal stigma surrounding professional grief expression HCPs lack sufficient training in preparation for patient deaths and providing palliative care
Institutional support and interventions AND coping with patient death (*n* = 1)						
Wenzel et al. (2011), *Oncology Nursing Forum* [43]	USA	Qualitative (focus groups)	Cross‐sectional	*n* = 34 (4–8 per group), oncology nurses	Work‐related bereavement, management of loss	Aim: Determine facilitators and barriers to managing patient loss from the perspectives of oncology nurses Challenges in managing patient loss: time constraints, emotionally high demands of field of work and collaborating with “outsiders” (of the field or the institution); closer attachment to patients seems to exacerbate feelings of grief in case of patient deaths Resources in coping with patient death: Individual (religion or faith, sports and “self‐care” [e.g., taking a bath]), team‐based (debriefing sessions and a strong team spirit in general) or rooted in the relationship with the patients and their families (e.g., feeling like one does “the best job” for the patients and their families) Organizational support for oncology nurses dealing with patient death seems to be insufficient, specific suggestions for improvements were made, ranging from “creating time and space for staff self‐care” (e.g., supporting and empathizing the importance of breaks) and providing collegial or professional support to improving efficiency in dealing with paperwork to allow nurses to spend more time with the dying patients and their families
All (*n* = 1)						
Plante & cyr (2011), *Paediatrics & Child Health* [44]	Canada	Quantitative (survey)^b^	Cross‐sectional	*n* = 101, mixed sample (46 nurses; 22 physicians; 11 residents; 13 respiratory therapists; 9 other)	Grief	Aim: Evaluate the intensity of grief experienced by HCPs after the death of a child and identify their needs and explore factors associated with a memorable patient death HCPs' grief: All HCPs (100%) expressed moderate to high levels of grief, with respiratory therapists and physicians reporting significantly higher levels than nurses (*M* = 29.4, SD = 4.1; *M* = 23.1, SD = 3.8 vs. *M* = 13.1, SD = 1.8); male gender and younger age was associated with higher grief scores while being comfortable with caring for a dying child was associated with a lower intensity of grief; being a parent or having lost a child one‐self, receiving previous training in palliative care or the length of the relationship with the child/the family were not associated with the intensity of the felt grief Factors influencing a memorable death: Unexpected death, length of relationship and affective bonding with the child/family, occurrence of death after palliative care or connected to a special event (e.g., Christmas), being personally involved in termination of treatment, feelings of guilt linked to the death or if the dying child had a twin Following a memorable death participants reported: Feelings of sadness, anger, relief, anxiety, sometimes guilt, the need to be alone, changes in appetite, trouble sleeping or more frequent mistakes at work, but also being more compassionate towards others 90% of sample successfully coped with patient deaths; positive coping strategies: Seeking emotional and social support (75%–85%, mostly with colleagues), positive reframing (80%), seeking distraction (57%), faith (55%) and humor (13%); potentially negative coping strategies: Self‐blame (40%), denial (6%), substance abuse (2%) and behavioral disengagement (1%) 40% wished for more support from their institution; suggestions were incoherent, but psychological counseling could be helpful as well as (voluntary) meetings after a patient's death, taking a break from work (even for 15–30 min) and further training in palliative care

*Note:* Assessing grief with questionnaire validated measure.

^a^ Texas revised inventory of grief, present scale (TRIG‐present).

^b^ Texas revised inventory of grief, both scales.

^c^ Grief experience inventory (GEI).

### 3.2 Synthesis of Results

Data charting revealed four main categories: “the experience of professional grief,” “influences on and of professional grief,” “coping with patient death,” and “institutional support and interventions.” Some studies addressed multiple categories, with varying numbers of studies per category (1–10, see Table 1).

#### 3.2.1 The Experience of Professional Grief

The first category explores the emotional dimensions of professional grief, examining intensity, frequency, effects and unique contextual factors. In response to patient deaths, HCPs experience a distinctive form of grief, encompassing typical expressions like crying and sadness, along with specific aspects such as guilt and helplessness [19, 21, 25, 36, 44]. This grief differs from personal grief due to the frequency of patient deaths in cancer care and the responsibility felt toward patients' lives, albeit with reduced intensity [21, 24, 41]. Studies show highly variable frequencies of professional grief, ranging from 23% to 100%, indicating multifactorial causes beyond patient deaths [8, 24, 25, 32, 44]. Factors making patient deaths challenging include the patient's age, closeness of the patient‐HCP relationship, unexpected outcomes, and family issues [11, 37, 38, 44]. HCPs also feel pressure to maintain composure, which can lead to personality changes and exhaustion [2, 5, 8, 26, 39]. However, positive outcomes include normalizing death, learning from patients, and enhancing life appreciation [25, 41].

### 3.3 Influences on and of Professional Grief

This category explores the relationship between professional grief and other side effects of caring for terminally ill patients. Positive associations between grief and compassion fatigue and burnout were found, and grief partially mediates the link between empathy and secondary traumatic stress. Social acknowledgment of grief affects levels of secondary traumatic stress [2, 3, 5, 26].

### 3.4 Coping With Patient Death

This category focuses on HCPs' approaches to managing patient loss and the extent to which these approaches aid in effective coping. HCPs employ various coping strategies, including seeking social support, relying on faith, engaging in hobbies, and using positive reframing. Barriers to coping include time constraints, heavy workloads, and gender‐related stereotypes. Some HCPs resort to detrimental strategies like emotional withdrawal or substance abuse [7, 28, 41, 44].

### 3.5 Institutional Support and Interventions

Institutions can mitigate the impact of patient losses by providing training, validation of grief, opportunities for breaks, and psychosocial support [38, 43]. Providing or fostering collegial support emerged as beneficial [38, 44]. Interventions such as grief debriefing sessions and peer support workshops have proven helpful [31, 32, 33].

#### 3.5.1 The Construct of Professional Grief

We assessed the existing terminology and definitions pertaining to professional grief. While the majority of articles uses terms like “grief” to depict distress following a patient's loss, few specifically mention “professional grief” ([29, 30]; see Table 1, nomenclature). Some use rather descriptive terms such as “psychological burden of a patient's death” or “work‐related bereavement” [29, 43]. Although “professional grief” was already coined in 1995 [20], subsequent research did not uniformly adopt this term. Furthermore, < 10% of articles provide definitions for professional grief (*n* = 3; see Supporting Information S3; [7, 8, 30]). These definitions generally align with the emotional impact of professional grief outlined above but vary in focus, with Granek et al. emphasizing oncologists' experiences [7, 8], while Treggalles and Lowrie consider professional grief within the broader context of grief in general [40]. Despite these efforts to define professional grief, consistent terminology and an established and comprehensive definition of the construct are lacking across the available literature.

## 4 Discussion

In this scoping review, we analyzed 34 studies on professional grief among HCPs in cancer care, primarily from the United States, Canada, and Israel, with limited evidence from other regions. Notably, a small number of research teams provided most of the available evidence on professional grief.

Qualitative studies focusing on nurses and physicians constitute the primary source of existing knowledge, reflecting their prevalence among HCPs and their significant engagement with patient care and death. This emphasis on nurses and physicians is understandable, as they represent the majority of healthcare professionals and are thus more likely to be directly affected by professional grief. However, evidence on other HCP groups like psychologists, social workers, and physical therapists is limited. Two studies suggest that psycho‐oncologists and respiratory therapists also experience significant grief levels [2, 44]. Future research should address a broader range of occupational roles in cancer care.

Just like general grief, professional grief can be a natural and adaptive response to loss, rather than inherently pathological. Current research on professional grief in cancer care hasn't distinctly separated it from general grief, reflecting the interconnectedness of these experiences. While the available evidence confirms that the distress HCPs may feel after a patient's death is a form of grief, there remains a lack of clarity regarding whether this specific type of grief is fundamentally different in essence or merely contextual, highlighting the need for a deeper understanding of its unique characteristics. Professional grief, in contrast to grief occurring over personal losses, involves frequent encounters with patient deaths, though not all patient deaths trigger it due to factors like the depth of the professional relationship. While grief is a universal response to loss, the nature of the relationship between the bereaved and the deceased likely shapes how it is experienced and expressed. When professional grief does arise, it tends to be less intense, possibly because it's confined to the workplace. Unlike the loss of a loved one, which typically disrupts multiple aspects of life and affects various personal, social, and emotional domains, professional grief may remain contained within the work environment, allowing healthcare professionals to maintain continuity in other areas of their lives [19, 24, 26]. Healthcare professionals encounter patient deaths within a structured, professional framework [45, 46], which may influence both the emotional impact and the way grief is expressed [2]. These differences suggest that contextual factors play a critical role and may contribute to distinguishing grief in a professional setting from grief in general. However, this link between grief and the workplace presents unique challenges, including a heightened sense of responsibility and occasional guilt over patient deaths. Additionally, healthcare professionals often perceive their professional grieving reactions as inappropriate or socially unacceptable (see Supporting Information S4 for an overview of contrasting elements of professional grief).

While most studies within the corpus of studies reviewed use terms like “grief,” few directly refer to “professional grief” or provide clear definitions (refer to Table 1, column: nomenclature for PG, for a comprehensive overview; 7,8,35). This inconsistent terminology poses a problem, introducing uncertainty in data interpretation.

Furthermore, the available quantitative studies have predominantly employed questionnaires tailored to the assessment of grief experienced within personal contexts [47, 48]. Nonetheless, the distinct nature of grief experienced in a professional context—presumably marked by elements such as guilt stemming from a sense of professional responsibility towards a patient's life—suggests that conventional assessment tools may fall short in comprehensively capturing professional grief. Therefore, while it is valid to emphasize that grief, regardless of context, is a human response to loss, further research is essential to better understand the specific characteristics and implications of *professional* grief.

### 4.1 Study Limitations

Our scoping review has several limitations. Firstly, our primary search was limited to three electronic databases, which may have caused us to overlook relevant publications. However, we prioritized search sensitivity, evident in the considerable number of abstracts reviewed. Secondly, this review focused solely on professional grief in cancer care. While HCPs in cancer care often face patient deaths, the distinction between them and those in other fields may not accurately reflect reality. It remains uncertain whether the fundamental construct or symptoms differ across disciplines. Therefore, it is possible that some pertinent articles were missed, particularly those not specific to cancer care but still relevant to understanding professional grief overall. Furthermore, there was a predominance of certain research groups and geographical locations, limiting generalizability. The absence of insights from the global south and Europe raises doubts about generalizability, despite including papers in English, German, and French. Although we recognize that there is a broader body of research on professional grief outside oncology, our focus on cancer care was guided by the need to explore this area, as it is one with relatively high patient mortality. We acknowledge that this focus may have led to the exclusion of some valuable studies relevant to professional grief in the broader field of medicine. However, our review serves as a foundational starting point to map relevant findings and research gaps in this specific context. Our review is the first of its kind to comprehensively address professional grief in cancer care, providing an inclusive overview of the current evidence and valuable insights for future investigations. Another strength is our interdisciplinary author team, including clinical expertise in oncology, palliative care, and psycho‐oncology, as well as extensive experience in literature reviews.

### 4.2 Clinical Implications

Despite the absence of a precisely defined construct and the variability in terminology, professional grief undeniably affects HCPs in cancer care. A common theme across reviewed studies is the acknowledgment that witnessing patient deaths may induce emotional distress in HCPs. HCPs express a need for structured guidance on managing patient deaths in cancer care [30]. The absence of such guidance may lead to adverse consequences such as compassion fatigue, burnout, and compromised care quality [8, 21, 24, 27]. Recognizing these challenges, it is crucial for institutions to explore which structures within medical school training and hospital settings could be most effective in integrating and normalizing the experience of grief as part of the work. Institutions may be falling short in their responsibility both to their employees and the patients they serve [24, 43, 44].

## 5 Conclusion

While the exact terminology and clear differentiation remain unclear, the presence of distress is evident and concerning. While some studies have explored contextual factors and effective management of what we might term as “professional grief,” comprehensive investigations are lacking. Future research should prioritize establishing a precise definition of “professional grief” and develop robust measures. Insights should be gathered from various occupations and geographic regions, within and beyond cancer care. These efforts are crucial for advancing towards a healthcare system that prioritizes the well‐being of its professionals.

## Author Contributions

Isabelle Scholl was the principal investigator of this study. Svenja Wandke and Isabelle Scholl both contributed to the design, conceptualization and preparation of the study. Data selection, extraction, analysis, and synthesis was primarily conducted by Svenja Wandke, and supported by Mareike Rutenkröger and Hannah Führes. Klaus Lang, Martin Härter and Karin Oechsle advised on conceptualization. All authors contributed to interpretation of results. Svenja Wandke drafted the manuscript. All authors critically revised the manuscript. The final version was approved by all authors.

## Conflicts of Interest

The authors declare no conflicts of interest.

## Supporting information

Supporting Information S1

Supporting Information S2

Supporting Information S3

Supporting Information S4

Supporting Information S5

## Data Availability

The authors have nothing to report.

## References

[1] E. Buglass , “Grief and Bereavement Theories,” Nursing Standard 24, no. 41 (June 2010): 44–47, 10.7748/ns2010.06.24.41.44.c7834. 20608339

[2] A. Engler‐Gross , G. Goldzweig , I. Hasson‐Ohayon , R. Laor‐Maayany , and M. Braun , “Grief Over Patients, Compassion Fatigue, and the Role of Social Acknowledgment Among Psycho‐Oncologists,” Psycho‐Oncology 29, no. 3 (2020): 493–499, 10.1002/pon.5286. 31713957

[3] R. Laor‐Maayany , G. Goldzweig , I. Hasson‐Ohayon , G. Bar‐Sela , A. Engler‐Gross , and M. Braun , “Compassion Fatigue Among Oncologists: The Role of Grief, Sense of Failure, and Exposure to Suffering and Death,” Supportive Care in Cancer 28, no. 4 (April 2020): 2025–2031, 10.1007/s00520-019-05009-3. 31392551 PMC7223813

[4] B. W. Corn , E. Shabtai , O. Merimsky , et al., “Do Oncologists Engage in Bereavement Practices? A Survey of the Israeli Society of Clinical Oncology and Radiation Therapy (ISCORT),” Oncologist 15, no. 3 (March 2010): 317–326, 10.1634/theoncologist.2009-0257. 20228130 PMC3227959

[5] H. Shi , B. Shan , J. Zheng , Y. Zhang , J. Zhang , and X. Hu , “Grief as a Mediator of the Relationship Between Empathy and Compassion Fatigue,” Psycho‐Oncology 31, no. 5 (2022): 840–847, 10.1002/pon.5875. 34997672

[6] H. Sung , J. Ferlay , R. L. Siegel , et al., “Global Cancer Statistics 2020: GLOBOCAN Estimates of Incidence and Mortality Worldwide for 36 Cancers in 185 Countries,” CA: A Cancer Journal for Clinicians 71, no. 3 (2021): 209–249, 10.3322/caac.21660. 33538338

[7] L. Granek , S. Ariad , S. Shapira , G. Bar‐Sela , and M. Ben‐David , “Barriers and Facilitators in Coping With Patient Death in Clinical Oncology,” Supportive Care in Cancer 24, no. 10 (October 2016): 4219–4227, 10.1007/s00520-016-3249-4. 27146494

[8] L. Granek , R. Tozer , P. Mazzotta , A. Ramjaun , and M. Krzyzanowska , “Nature and Impact of Grief Over Patient Loss on Oncologists’ Personal and Professional Lives,” Archives of Internal Medicine 172, no. 12 (June 2012): 964–966, 10.1001/archinternmed.2012.1426. 22732754

[9] L. B. P. A. D. Santos , W. D. A. Alvarenga , A. C. A. B. Leite , et al., “Compassion Fatigue: A Comprehensive Discussion on Its Development and Repercussions Among Oncology Nurses,” Seminars in Oncology Nursing 40, no. 4 (August 2024): 151655, 10.1016/j.soncn.2024.151655. 38782693

[10] J. Banks , V. Lopez , A. Sahay , and M. Cleary , “A Scoping Review of Compassion Fatigue Among Oncology Nurses Caring for Adult Patients,” Cancer Nursing 47, no. 4 (July 2024): E213–E225, 10.1097/ncc.0000000000001226. 36944157

[11] L. Granek , M. K. Krzyzanowska , R. Tozer , and P. Mazzotta , “Difficult Patient Loss and Physician Culture for Oncologists Grieving Patient Loss,” Journal of Palliative Medicine 15, no. 11 (November 2012): 1254–1260, 10.1089/jpm.2012.0245. 23016965

[12] M. K. Eichorst , A. L. Fromenthal , G. M. Harris , C. D. Reel , and R. S. Allen , “In the Presence of Death and Dying: Death Attitudes and Compassion Fatigue Among Certified Nursing Assistants in Skilled Care,” Aging & Mental Health (September 2024): 1–10, 10.1080/13607863.2024.2399089. 39244655

[13] H. Ibrahim , L. Oyoun Alsoud , K. West , et al., “Interventions to Support Medical Trainee Well‐Being After Patient Death: A Scoping Review,” Journal of Hospital Medicine 19, no. 11 (November 2024): 1044–1052, 10.1002/jhm.13489. 39154261

[14] A. Sinnathamby , H. Ibrahim , Y. T. Ong , et al., “Towards a Theory of Compassion Fatigue in Palliative Care and Oncology: A Systematic Scoping Review,” American Journal of Hospice and Palliative Medicine® (January 2025): 10499091251315183. 39825792 10.1177/10499091251315183 PMC12705876

[15] A. C. Tricco , E. Lillie , W. Zarin , et al., “PRISMA Extension for Scoping Reviews (PRISMA‐ScR): Checklist and Explanation,” Annals of Internal Medicine 169, no. 7 (October 2018): 467–473, 10.7326/m18-0850. 30178033

[16] H. Arksey and L. O’Malley , “Scoping Studies: Towards a Methodological Framework,” International Journal of Social Research Methodology 8, no. 1 (February 2005): 19–32, 10.1080/1364557032000119616.

[17] M. Ouzzani , H. Hammady , Z. Fedorowicz , and A. Elmagarmid , “Rayyan—A Web and Mobile App for Systematic Reviews,” Systematic Reviews 5, no. 1 (December 2016): 210, 10.1186/s13643-016-0384-4. 27919275 PMC5139140

[18] N. G. Chau , C. Zimmermann , C. Ma , N. Taback , and M. K. Krzyzanowska , “Bereavement Practices of Physicians in Oncology and Palliative Care,” Archives of Internal Medicine 169, no. 10 (May 2009): 963–971, 10.1001/archinternmed.2009.118. 19468090

[19] T. M. Conte , “The Lived Experience of Work‐Related Loss and Grief Among Pediatric Oncology Nurses,” Journal of Hospice and Palliative Nursing 16, no. 1 (February 2014): 40–46, 10.1097/njh.0000000000000019.

[20] M. A. Feldstein and P. G. Buschman , “Oncology Nurses and Chronic Compounded Grief,” Cancer Nursing 18, no. 3 (June 1995): 228. 7600555

[21] L. Granek , U. Bartels , K. Scheinemann , M. Labrecque , and M. Barrera , “Grief Reactions and Impact of Patient Death on Pediatric Oncologists,” Pediatric Blood and Cancer 62, no. 1 (2015): 134–142, 10.1002/pbc.25228. 25214471

[22] L. Granek , S. Ariad , O. Nakash , M. Cohen , G. Bar‐Sela , and M. Ben‐David , “Mixed‐Methods Study of the Impact of Chronic Patient Death on Oncologists’ Personal and Professional Lives,” Journal of Oncology Practice 13, no. 1 (January 2017): e1–e10, 10.1200/jop.2016.014746. 28084882

[23] L. Granek , M. Ben‐David , S. Shapira , G. Bar‐Sela , and S. Ariad , “Grief Symptoms and Difficult Patient Loss for Oncologists in Response to Patient Death,” Psycho‐Oncology 26, no. 7 (2017): 960–966, 10.1002/pon.4118. 26988940

[24] L. J. Kaplan , “Toward a Model of Caregiver Grief: Nurses’ Experiences of Treating Dying Children,” OMEGA ‐ Journal of Death and Dying 41, no. 3 (November 2000): 187–206, 10.2190/ngg6-ypah-40ab-cnx0.

[25] A. Kaur , M. P. Sharma , and S. K. Chaturvedi , “Felt Needs of Cancer Palliative Care Professionals Working in India: A Qualitative Study,” Indian Journal of Palliative Care 27, no. 4 (2021): 544–551, 10.25259/ijpc_125_21. 34898950 PMC8655635

[26] G. Hayuni , I. Hasson‐Ohayon , G. Goldzweig , G. Bar Sela , and M. Braun , “Between Empathy and Grief: The Mediating Effect of Compassion Fatigue Among Oncologists,” Psycho‐Oncology 28, no. 12 (2019): 2344–2350, 10.1002/pon.5227. 31518033

[27] L. Granek , P. Mazzotta , R. Tozer , and M. K. Krzyzanowska , “Oncologists’ Protocol and Coping Strategies in Dealing With Patient Loss,” Death Studies 37, no. 10 (November 2013): 937–952, 10.1080/07481187.2012.692461. 24517522

[28] L. Granek , M. Barrera , K. Scheinemann , and U. Bartels , “Pediatric Oncologists’ Coping Strategies for Dealing With Patient Death,” Journal of Psychosocial Oncology 34, no. 1–2 (March 2016): 39–59, 10.1080/07347332.2015.1127306. 26865337

[29] M. Lee , K. Choe , S. Kim , and Y. Shim , “How Do Oncology Nurses Cope With the Psychological Burden of Caring for Dying Patients?,” Cancer Nursing 46, no. 4 (July 2023): E245–E252, 10.1097/ncc.0000000000001102. 35245226

[30] L. Granek , P. Mazzotta , R. Tozer , and M. K. Krzyzanowska , “What Do Oncologists Want?,” Supportive Care in Cancer 20, no. 10 (October 2012): 2627–2632, 10.1007/s00520-012-1528-2. 22714702

[31] P. S. Hinds , P. Puckett , M. Donohoe , et al., “The Impact of a Grief Workshop for Pediatric Oncology Nurses on Their Grief and Perceived Stress,” Journal of Pediatric Nursing 9, no. 6 (December 1994): 388–397. 7837057

[32] E. A. Keene , N. Hutton , B. Hall , and C. Rushton , “Bereavement Debriefing Sessions: An Intervention to Support Health Care Professionals in Managing Their Grief After the Death of a Patient,” Pediatric Nursing 36, no. 4 (2010). 20860257

[33] C. F. Macpherson , “Peer‐Supported Storytelling for Grieving Pediatric Oncology Nurses,” Journal of Pediatric Oncology Nursing 25, no. 3 (May 2008): 148–163, 10.1177/1043454208317236. 18413700

[34] A. E. Rettig , E. Lambrecht‐Stock , K. Bohley , et al., “Remembrance and Renewal: Health Care Staff Spiritual Self‐Care,” Journal of Holistic Nursing 38, no. 1 (March 2020): 139–146, 10.1177/0898010119900412.

[35] A. E. Rettig , E. Lambrecht‐Stock , A. Lindsey , and L. T. Sinnott , “Describing Remembrance & Renewal: A Holistic Self‐Care Program,” Journal of Holistic Nursing 41, no. 4 (December 2023): 327–334. 36945872 10.1177/08980101231163448

[36] Y. J. M. Chew , S. L. L. Ang , and S. Shorey , “Experiences of New Nurses Dealing With Death in a Paediatric Setting: A Descriptive Qualitative Study,” Journal of Advanced Nursing 77, no. 1 (2021): 343–354, 10.1111/jan.14602. 33074568

[37] L. Granek , L. Barbera , O. Nakash , M. Cohen , and M. K. Krzyzanowska , “Experiences of Canadian Oncologists With Difficult Patient Deaths and Coping Strategies Used,” Current Oncology 24, no. 4 (August 2017): 277–284, 10.3747/co.24.3527. PMC5576467 28874898

[38] L. Granek , M. Ben‐David , O. Nakash , et al., “Oncologists’ Negative Attitudes Towards Expressing Emotion Over Patient Death and Burnout,” Supportive Care in Cancer 25, no. 5 (May 2017): 1607–1614, 10.1007/s00520-016-3562-y. 28084531

[39] D. Puente‐Fernández , M. M. Lozano‐Romero , R. Montoya‐Juárez , C. Martí‐García , C. Campos‐Calderón , and C. Hueso‐Montoro , “Nursing Professionals’ Attitudes, Strategies, and Care Practices Towards Death: A Systematic Review of Qualitative Studies,” Journal of Nursing Scholarship 52, no. 3 (2020): 301–310, 10.1111/jnu.12550. 32190978

[40] K. Treggalles and D. Lowrie , “An Exploration of the Lived Experience of Professional Grief Among Occupational Therapists Working in Palliative Care Settings,” Australian Occupational Therapy Journal 65, no. 4 (2018): 329–337, 10.1111/1440-1630.12477. 29797520

[41] S. C. Zambrano , A. Chur‐Hansen , and G. B. Crawford , “The Experiences, Coping Mechanisms, and Impact of Death and Dying on Palliative Medicine Specialists,” Palliative & Supportive Care 12, no. 4 (August 2014): 309–316, 10.1017/s1478951513000138. 23750857

[42] J. Gomes Maia De Sena , C. De Freitas Melo , A. Vieira De Vasconcelos , L. Cavalcante Teixeira , E. Miessa Ruiz , and R. S. Fernandes Alves , “The Care for Oncologic Patients Undergoing Pediatric Palliative Care and the Griefs of a Health Team,” Psicooncología 20, no. 1 (April 2023): 103–119, 10.5209/psic.78677.

[43] J. Wenzel , M. Shaha , R. Klimmek , and S. Krumm , “Working Through Grief and Loss: Oncology Nurses’ Perspectives on Professional Bereavement,” Oncology Nursing Forum 38, no. 4 (July 2011): E272–E282, 10.1188/11.onf.e272-e282. 21708522 PMC4648272

[44] J. Plante and C. Cyr , “Health Care Professionals’ Grief After the Death of a Child,” Paediatrics and Child Health 16, no. 4 (April 2011): 213–216, 10.1093/pch/16.4.213. 22468124 PMC3076172

[45] M. Shutzberg , “The Doctor as Parent, Partner, Provider… or Comrade? Distribution of Power in Past and Present Models of the Doctor–Patient Relationship,” Health Care Analysis 29, no. 3 (September 2021): 231–248, 10.1007/s10728-021-00432-2. 33905025 PMC8322008

[46] World Medical Association . “WMA International Code of Medical Ethics,” (2022).

[47] T. Faschingbauer , The Texas Inventory of Grief–Revised (Honeycomb Publishing, 1981).

[48] C. M. Sanders , P. A. Mauger , and P. N. Strong , A Manual for the Grief Experience Inventory (Consulting Psychologists Press, 1985), 31.

