# Supplementary material for: Professional Grief in Cancer Care—A Scoping Review
Source: Psychooncology. 2025 Apr 25;34(5):e70156. doi: 10.1002/pon.70156 (PMC12031695; doi:10.1002/pon.70156)
Supplement: Supplementary file 1 — Supporting Information S1 [file PON-34-e70156-s003.docx]

**Supplementary File 1:** **Professional Grief – A scoping review**

Checklist: Inclusion & Exclusion Criteria

Note: For a publication to be included in the review, all inclusion criteria must apply, and none of the exclusion criteria must apply.

**General**

| Criterion | Inclusion (✔ Required) | Exclusion (✘ Not Allowed) |
| --- | --- | --- |
| Language | ☐ German, English, or French | ☐ Other languages |
| Full-text availability | ☐ Available | ☐ Not available |

**Population**

| Criterion | Inclusion (✔ Required) | Exclusion (✘ Not Allowed) |
| --- | --- | --- |
| Healthcare Professionals (HCP) | ☐ Doctors, Psychologists, Nurses, Social Workers, and other graduated professionals involved in medical or paramedical care | ☐ Administrative staff, students (medicine, nursing, or other HCP in training) |

**Event**

| Criterion | Inclusion (✔ Required) | Exclusion (✘ Not Allowed) |
| --- | --- | --- |
| Nature of Event | ☐ A patient’s death | ☐ Death of a partner, friend, relative, colleague, or supervisor |

**Concept of Interest**

| Criterion | Inclusion (✔ Required) | Exclusion (✘ Not Allowed) |
| --- | --- | --- |
| Main Focus | ☐ Professional grief, including reactions to or coping with a patient’s death as one of or the primary outcome(s) | ☐ Other constructs as primary outcomes (e.g., burnout, compassion fatigue) |

**Context**

| Criterion | Inclusion (✔ Required) | Exclusion (✘ Not Allowed) |
| --- | --- | --- |
| Medical Setting | ☐ Cancer care, including medical, paramedical, psychotherapeutic, or social work contexts (e.g., oncology, radiology, hemato-oncology) | ☐ Unspecified context, mixed patient populations (cancer care + other), other settings (e.g. intensive care units) |

**Methodology**

| Criterion | Inclusion (✔ Required) | Exclusion (✘ Not Allowed) |
| --- | --- | --- |
| Study Type | ☐ Empirical research (qualitative, quantitative, or mixed methods), systematic literature syntheses | ☐ Non-empirical studies, personal narratives, opinion pieces |
