# Supplementary material for: Professional Grief in Cancer Care—A Scoping Review
Source: Psychooncology. 2025 Apr 25;34(5):e70156. doi: 10.1002/pon.70156 (PMC12031695; doi:10.1002/pon.70156)
Supplement: Supplementary file 2 — Supporting Information S2 [file PON-34-e70156-s005.docx]

Supplementary file 2, Example for a search strategy

The final search strategy for MEDLINE was the following: ("Health care professionals" OR "medical professionals" OR psychologist OR psychooncologist OR oncologist OR (healthcare provider[MeSH Terms]) OR (health personnel[MeSH Terms])) AND (Oncology OR Cancer OR Psychooncology OR (oncology[MeSH Terms])) AND („patient death" OR “patient loss” OR death) AND (grief[MeSH Terms] OR "professional grief" OR "staff grief" OR (bereavement[MeSH Terms]) OR "professional bereavement" OR grief support OR "grief education" OR coping OR (coping death[MeSH Terms]) OR mourning)
