# Supplementary material for: Professional Grief in Cancer Care—A Scoping Review
Source: Psychooncology. 2025 Apr 25;34(5):e70156. doi: 10.1002/pon.70156 (PMC12031695; doi:10.1002/pon.70156)
Supplement: Supplementary file 4 — Supporting Information S4 [file PON-34-e70156-s002.docx]

Supplementary file 4, Contrasting professional grief and grief in general

| **Grief (in general)** | vs. | **Professional Grief** |
| --- | --- | --- |
| Infrequent deaths |  | Common (everyday) Deaths |
| Occurs highly likely after each loss |  | Does not occur necessarily after every loss (Frequency fluctuations between 23% and 100%) |
| Fundamentally impactful losses |  | Losses affect only one area of life |
| High intensity of distress |  | Moderate to high intensity of distress |
| No responsibility for the dying process |  | Shared responsibility for the dying process, leading to feelings of guilt |
| Perceived as valid grief by mourners |  | Perceived as disenfranchised Grief? ("need to 'keep it together'") |
