# Supplementary material for: Professional Grief in Cancer Care—A Scoping Review
Source: Psychooncology. 2025 Apr 25;34(5):e70156. doi: 10.1002/pon.70156 (PMC12031695; doi:10.1002/pon.70156)
Supplement: Supplementary file 5 — Supporting Information S5 [file PON-34-e70156-s004.docx]

Supplementary file 5, List of records in full-text screening with reasons for exclusion

|  | Author(s) | Title | Year | Journal | Exclusion/Inclusion | Reason for Exclusion |
| --- | --- | --- | --- | --- | --- | --- |
|  | AJ Adelman | The hand of fate: On mourning the death of a patient. | 2013 | The Therapist in Mourning: From the Faraway Nearby | Excluded | Methodology |
|  | A. Agnew et al. | Bereavement needs assessment in specialist palliative care: a review of the literature. | 2010 | Palliative Medicine | Excluded | Concept of interest |
|  | A. Alvarez-del-Rio et al. | Experience of oncology residents with death: A qualitative study in Mexico. | 2019 | BMC Medical Ethics | Excluded | Population |
|  | A. Amir, GP. Kalemkerian | Run for your life: the reaction of some professionals to a person with cancer. | 2003 | Journal of Clinical Oncology | Excluded | Methodology |
|  | E. Anderson, J. Sandars, D. Klinnair | The nature and benefits of team-based reflection on a patient death by healthcare professionals: A scoping review. | 2019 | Journals of Interprofessional Care | Excluded | Context |
|  | NE Anderson, B. kent, RG Owens | Experiencing patient death in clinical practice: Nurses' recollections of their earliest memorable patient death. | 2015 | International Journal of Nursing Studies | Excluded | Context |
|  | MA Ashby et al. | An enquiry into death and dying at the Adelaide Children's Hospital: a useful model? | 1991 | The Medical Journal of Australia | Excluded | Context |
|  | A. Athanasios | Do doctors grieve: Addressing physician grief and cultivating better doctors. | 2020 | Pastoral Psychology | Excluded | Methodology |
|  | L. Baider, S. Wein | Reality and fugues in physicians facing death: confrontation, coping, and adaptation at the bedside. | 2001 | Critical Reviews in Oncology/Hematology | Excluded | Methodology |
|  | D. Bainbridge et al. | The care experiences of patients who die in residential hospice: A qualitative analysis of the last three months of life from the views of bereaved caregivers. | 2018 | Palliative & Supportive care | Excluded | Population |
|  | JH Baker et al. | Implementation and evaluation of an automated Patient Death Notification policy at a tertiary pediatric oncology referral center. | 2011 | Journal of Pain and Symptom Management | Excluded | Concept of interest |
|  | D. Barham | The last 48 hours of life. | 2002 | Contemporary Nurse | Excluded | Methodology |
|  | S. Barnes et al. | Health professionals' experiences of grief associated with the death of pediatric patients: A systematic review. | 2020 | JBI Evidence Synthesis | Excluded | Context |
|  | D. Barton et al. | Death and dying: a course for medical students. | 1972 | Journal of Medical Education | Excluded | Population |
|  | R. Bauer-Mehren, K. Kopp-Breinlinger, P. Rechenberg-Winter | Kaleidoskop der Trauer | 2003 |  | Excluded | Concept of interest |
|  | S. Beardsmore, N. Fitzmaurice | Palliative care in paediatric oncology. | 2002 | European Journal of Cancer | Excluded | Concept of interest |
|  | E. Becze | To retain oncology nurses, offer grief resolution. | 2012 | ONS Connect | Excluded | Methodology |
|  | HU Bender | A big pot of colored sweets. | 2015 | Journal of Palliative Medicine | Excluded | Methodology |
|  | GF Blackall | Still. | 2013 | JAMA Pediatrics | Excluded | Methodology |
|  | SD Block, JA Billings | Learning from the dying. | 2005 | The New England Journal of Medicine | Excluded | Methodology |
|  | GA Bonanno, S. Kaltman | Toward an integrative perspective on bereavement. | 1999 | Psychological Bulletin | Excluded | Concept of interest |
|  | R. Borscheid | Essay on Being a Doctor: Patient Mentor. | 2018 | Methodist DeBakey Cardiovascular Journal | Excluded | Methodology |
|  | A. Bovero et al. | Medical Students Reflections Toward End-of-Life: a Hospice Experience. | 2018 | Journal of Cancer Education | Excluded | Population |
|  | M. Braun, D. Gordon, B. Uziely | Associations between oncology nurses' attitudes toward death and caring for dying patients. | 2010 | Oncology Nursing Forum | Excluded | Concept of interest |
|  | M. Braun et al. | The paradox: guilt as an antidote to helplessness among oncologists. | 2022 | Supportive Care in Cancer | Excluded | Concept of interest |
|  | LJ Breen et al. | The "specter" of cancer: Exploring secondary trauma for health professionals providing cancer support and counseling. | 2014 | Psychological Services | Excluded | Concept of interest |
|  | M. Browall et al. | Existential encounters: nurses' descriptions of critical incidents in end-of-life cancer care. | 2014 | European Journal of Oncology Nursing | Excluded | Concept of interest |
|  | G. Brown | Coping with a patient's death. | 2017 | Nursing Standard | Excluded | Methodology |
|  | C. Burke, SM Gerraughty | An oncology unit's initiation of a bereavement support program. | 1994 | Oncology Nursing Forum | Excluded | Concept of interest |
|  | RN Butler et al. | Palliative medicine: providing care when cure is not possible. A roundtable discussion: Part I. | 1996 | Geriatrics | Excluded | Methodology |
|  | C. Cannon | Death happens every day. | 2013 | Oncology Nursing Forum | Excluded | Methodology |
|  | D. Casarett, JS Kutner, J. Abrahm | Life after death: a practical approach to grief and bereavement | 2001 | Annals of Internal Medicine | Excluded | Event |
|  | BJ Cashavelly et al. | The forgotten team member: meeting the needs of oncology support staff. | 2008 | The Oncologist | Excluded | Population |
|  | C. Catania, V. Zagonel | Care and self-care/god as a mirror of the self. | 2009 | Journal of Cancer Education | Excluded | Methodology |
|  | ER Carton, JE Hupcey | The forgotten mourners: Addressing health care provider grief. | 2014 | Journal of Hospice & Palliative Nursing | Excluded | Context |
|  | AP Caton, P. Klemm | Introduction of novice oncology nurses to end-of-life care. | 2006 | Clinical Journal of Oncology Nursing | Excluded | Concept of interest |
|  | B. Cevik, S. Kav | Attitudes and experiences of nurses toward death and caring for dying patients in Turkey. | 2013 | Cancer Nursing | Excluded | Context |
|  | HY Chan, LH Chan, CW Chan | The perceptions and experiences of nurses and bereaved families towards bereavement care in an oncology unit. | 2013 | Supportive Care in Cancer | Excluded | Concept of interest |
|  | WP Chang | How social support affects the ability of clinical nursing per-sonnel to cope with death. | 2013 | Applied Nursing Research | Excluded | Context |
|  | S. Chanock | Reflections on events surrounding the time of diagnosis in pediatric oncology. | 2001 | Journal of Pediatric Hematology/ Oncology | Excluded | Methodology |
|  | NG Chau et al. | Bereavement practices of physicians in oncology and palliative care. | 2009 | Archives of Internal Medicine | Included | / |
|  | C. Chen et al. | The meaning of patient deaths for professional care-givers: A quantitative construct validation. | 2023 | Death Studies | Excluded | Context |
|  | C. Chen et al. | The meaning of patient deaths for professional caregivers: A quantitative construct validation. | 2022 | Death Studies | Excluded | Context |
|  | C. Chen et al. | Professional caregivers' bereavement after patients' deaths: A scoping review of quantitative studies. | 2019 | Death Studies | Excluded | Context |
|  | C. Chen et al. | Bereavement process of professional caregivers after deaths of their patients: A meta-ethnographic synthesis of qualitative studies and an integrated model. | 2018 | International Journal of Nursing Studies | Excluded | Context |
|  | C. Chen et al. | Gender differences and the effects of bereavement-related psychological distress in health outcomes | 1999 | Psychological Medicine | Excluded | Event |
|  | C. Cherny, N. Coyle, KM Foley | Guidelines in the care of the dying cancer patient. | 1996 | Hematology/ Oncology Clinics of North America | Excluded | Concept of interest |
|  | YJM Chew, SLL Ang, S. Shorey | Experiences of new nurses dealing with death in a paediatric setting: A descriptive qualitative study. | 2021 | Journal of Advanced Nursing | Included | / |
|  | PJ Clayton, HS Darvish | Course of depressive symptoms following the stress of bereavement | 1979 | Stress and mental disorder | Excluded | Concept of interest |
|  | EM Clingerman | Bereavement tasks for nursing students. | 1996 | Nurse educator | Excluded | Population |
|  | S. Collins-Tracey et al. | Contacting bereaved relatives: The views and practices of palliative care and oncology health care professionals. | 2009 | Journal of Pain and Symptom Management | Excluded | Concept of interest |
|  | TM Conte | The lived experience of work‐related loss and grief among pediatric oncology nurses | 2014 | Journal of Hospice & Palliative Nursing | Included | / |
|  | D. Coody | High expectations. Nurses who work with children who might die. | 1985 | Nursing Clinics of North America | Excluded | Methodology |
|  | G. Copp | A review of current theories of death and dying. | 1998 | Journal of Advanced Nursing | Excluded | Concept of interest |
|  | BW Corn et al. | Do oncologists engage in bereavement practices? A survey of the Israeli Society of Clinical Oncology and Radiation Therapy (ISCORT). | 2010 | The Oncologist | Included | / |
|  | E. Corruble, R. Pies, S. Zisook | Grief in health care professionals: when screening for major depression is needed. | 2012 | Archives of Internal Medicine | Excluded | Context |
|  | A. Couldrick | Optimizing bereavement outcome: reading the road ahead. | 1992 | Social Science & Medicine | Excluded | Methodology |
|  | KV Cowles, BL Rodgers | The concept of grief: A foundation for nursing research and practice | 1991 | Research in Nursing & Health | Excluded | Event |
|  | L. Cramond et al. | Experiences of clinical psychologists working in palliative care: A qualitative study. | 2020 | European Journal of Cancer Care | Excluded | Event |
|  | C. Crowe et al. | Self-care and burnout in oncology professionals. | 2016 | Cancer: Treatment, decision making and Quality of life. | Excluded | Concept of interest |
|  | C. Crowe et al. | Burnout and self-care considerations for oncology professionals. | 2015 | Journal of Pain Management | Excluded | Concept of interest |
|  | L. Croxon, L. Deravin, J. Anderson | Dealing with end of life—New graduated nurse experiences | 2018 | Journal of Clinical Nursing | Excluded | Context |
|  | MK Das et al. | Perceptions of the parents of deceased children and of healthcare providers about end-of-life communication and breaking bad news at a tertiary care public hospital in India: A qualitative exploratory study. | 2021 | PLoS One | Excluded | Context |
|  | KW Davidson | Social work with cancer patients: stresses and coping patterns. | 1985 | Social Work in Health Care | Excluded | Context |
|  | KW Davidson & Z. Foster | Social work with dying and bereaved clients: helping the workers. | 1995 | Social Work in Health Care | Excluded | Concept of interest |
|  | B. Davies et al. | Caring for dying children: nurses' experiences | 1996 | Pediatric Nursing | Excluded | Context |
|  | S. Dein, SQ Abbas | The stresses of volunteering in a hospice: a qualitative study. | 2005 | Palliative Medicine | Excluded | Population |
|  | AC Delafontaine et al. | Impact of confrontation to patient suffering and death on wellbeing and burnout in professionals: a cross-sectional study | 2024 | BMC Palliative Care | Excluded | Concept of interest |
|  | N. Delvaux, D. Razavi, C. Farvacques | Cancer care: A stress for health professionals. | 1988 | Social Science & Medicine | Excluded | Methodology |
|  | D. Dixon et al. | Mechanisms of Support: Coping with Loss in a Major Children's Hospital. | 2005 | Social Work in Health Care | Excluded | Context |
|  | A. Doron, S. Mendlovic | Dying in the psychiatric ward. | 2008 | The Israel journal of psychiatry and related sciences | Excluded | Context |
|  | ML Dwyer et al. | Death is a part of life: Considerations for the natural death of a therapy patient. | 2012 | Professional Psychology: Research and Practice | Excluded | Event |
|  | M. Edo-Gual et al. | The impact of death and dying on nursing students: An explanatory model. | 2014 | Journal of Clinical Nursing | Excluded | Population |
|  | S. Eggly et al. | Physicians' conceptualization of "closure" as a benefit of physician-parent follow-up meetings after a child's death in the pediatric intensive care unit. | 2013 | Journal of Palliative Care | Excluded | Context |
|  | M. Ekedahl, Y. Wengstrom | Nurses in cancer care - stress when encountering existential issues. | 2007 | European Journal of Oncology Nursing | Excluded | Concept of interest |
|  | A. El-Jawahri et al. | Psychological distress in bereaved caregivers of patients with advanced cancer. | 2021 | Journal of Pain and Symptom Management | Excluded | Population |
|  | EJ Emanuel et al. | Talking with terminally ill patients and their caregivers about death, dying, and bereavement: Is it stressful? Is it helpful? | 2004 | Archives of internal medicine | Excluded | Event |
|  | J. Eng et al. | Patient Death Debriefing Sessions to Support Residents' Emotional Reactions to Patient Deaths. | 2015 | Journal of graduate medical education | Excluded | Context |
|  | A. Engler-Gross et al. | Grief over patients, compassion fatigue, and the role of social acknowledgment among psycho-oncologists. | 2020 | Psycho-Oncology | Included | / |
|  | MA Feldstein, PB Gemma | Oncology nurses and chronic compounded grief. | 1995 | Cancer Nursing | Included | / |
|  | B. Ferrell et al. | Analysis of content regarding death and bereavement in nursing texts. | 1999 | Psycho-Oncology | Excluded | Population |
|  | D. Field | Special not different: general practitioners' accounts of their care of dying people. | 1998 | Social Science & Medicine | Excluded | Context |
|  | MI Fitch, D. Bakker, M. Conlon | Important issues in clinical practice: perspectives of oncology nurses. | 1999 | Canadian Oncology Nursing Journal | Excluded | Concept of interest |
|  | GA Florio, JP Donnelly, MA Zevon | The structure of work-related stress and coping among oncology nurses in high-stress medical settings: a transactional analysis. | 1998 | Journal of Occupational Health Psychology | Excluded | Concept of interest |
|  | GV Foley, EH Whittam | Care of the child dying of cancer: Part II. | 1991 | CA: A Cancer Journal of Clinicians | Excluded | Methodology |
|  | DR Freyer | This work we do: reflections from a pediatric hematology/oncology memorial service. | 2001 | Journal of Pediatric Hematology/Oncology | Excluded | Methodology |
|  | LM Funk et al. | The emotional labor of personal grief in palliative care: Balancing caring and professional identities. | 2017 | Qualitative Health Research | Excluded | Context |
|  | G. Gama, F. Barbosa, M. Vieira | Personal determinants of nurses' burnout in end of life care. | 2014 | European Journal of Oncology Nursing | Excluded | Concept of interest |
|  | JL Genevro, TL Miller | The Emotional and Economic Costs of Bereavement in Health Care Settings | 2010 | Psychologica Belgica | Excluded | Methodology |
|  | JL Genevro, T. Marshall, T. Miller | Report on bereavement and grief research | 2004 | Death Studies | Excluded | Methodology |
|  | LA Gerber | Transformations in self-understanding in surgeons whose treatment efforts were not successful. | 1990 | American Journal of Psychotherapy | Excluded | Event |
|  | L. Gerow et al. | Creating a curtain of protection: Nurses' experiences of grief following patient death. | 2010 | Journal of Nursing Scholarship | Excluded | Context |
|  | G. Giddings | The ties that bind: a reflection on physician grief. | 2010 | Supportive Care in Cancer | Excluded | Methodology |
|  | KR Gilbert | Taking a narrative approach to grief research: Finding meaning in stories | 2002 | Death studies | Excluded | Methodology |
|  | T. Gilewski | The art of medicine: teaching oncology fellows about the end of life. | 2001 | Critical reviews in Oncology/Hematology | Excluded | Methodology |
|  | J. Goertzen | Death of a child. | 1993 | Canadian Family Physician | Excluded | Methodology |
|  | I. Gorfinkel, M. Bernstein | House calls: what doctors get when they give. | 2020 | CMAJ: Canadian Medical Association Journal | Excluded | Methodology |
|  | L. Granek et al. | Oncologists' communication about end of life: The relationship among secondary traumatic stress, compassion satisfaction, and approach and avoidance communication. | 2017 | Psycho-Oncology | Excluded | Concept of interest |
|  | L. Granek et al. | Grief symptoms and difficult patient loss for oncologists in response to patient death. | 2017 | Psycho-Oncology | Included | / |
|  | L. Granek et al. | Experiences of Canadian oncologists with difficult patient deaths and coping strategies used. | 2017 | Current Oncology | Included | / |
|  | L. Granek et al. | Oncologists' negative attitudes towards expressing emotion over patient death and burnout. | 2017 | Supportive care in Cancer | Included | / |
|  | L. Granek et al. | Mixed-Methods Study of the Impact of Chronic Patient Death on Oncologists' Personal and Professional Lives. | 2017 | Journal of Oncology Practice | Included | / |
|  | L. Granek et al. | Pediatric oncologists' coping strategies for dealing with patient death. | 2016 | Journal of Psychosocial Oncology | Included | / |
|  | L. Granek et al. | Barriers and facilitators in coping with patient death in clinical oncology. | 2016 | Supportive Care in Cancer | Included | / |
|  | L. Granek et al. | Gender differences in the effect of grief reactions and burnout on emotional distress among clinical oncologists. | 2016 | Cancer | Excluded | Concept of interest |
|  | L. Granek et al. | Challenges faced by pediatric oncology fellows when patients die during their training. | 2015 | Journal of Oncology Practice | Excluded | Population |
|  | L. Granek et al. | When a child dies: Pediatric oncologists' follow-up practices with families after the death of their child. | 2015 | Psycho-Oncology | Excluded | Concept of interest |
|  | L. Granek et al. | Challenging patient deaths in pediatric oncology. | 2015 | Supportive Care in Cancer | Excluded | Concept of interest |
|  | L. Granek et al. | Grief reactions and impact of patient death on pediatric oncologists. | 2015 | Pediatric Blood & Cancer | Included | / |
|  | L. Granek et al. | Oncologists' protocol and coping strategies in dealing with patient loss. | 2013 | Death Studies | Included | / |
|  | L. Granek et al. | Difficult patient loss and physician culture for oncologists grieving patient loss. | 2012 | Journal of Palliative Medicine | Included | / |
|  | L. Granek et al. | What do oncologists want? Suggestions from oncologists on how their institutions can support them in dealing with patient loss. | 2012 | Supportive Care in Cancer | Included | / |
|  | L. Granek et al. | Nature and impact of grief over patient loss on oncologists' personal and professional lives. | 2012 | Archives of Internal Medicine | Included | / |
|  | L. Granek | Grief as pathology: The evolution of grief theory in psychology from Freud to the present. | 2010 | History of Psychology | Excluded | Concept of interest |
|  | A. Grech, J. Depares, J. Scerri | Being on the Frontline: Nurses' Experiences Providing End-of-Life Care to Adults With Hematologic Malignancies. | 2018 | Journal of Hospice and Palliative Nursing | Excluded | Concept of Interest |
|  | WJC Grove | Remembering patients who die: Exploring the meaning conveyed in notes to the researcher. | 2008 | Illness, Crisis & Loss | Excluded | Context |
|  | Q. Guo, R. Zheng | Assessing oncology nurses' attitudes towards death and the prevalence of burnout: A cross-sectional study. | 2019 | European Journal of Oncology Nursing | Excluded | Event |
|  | G. Hayuni et al. | Between empathy and grief: The mediating effect of compassion fatigue among oncologists. | 2019 | Psycho-Oncology | Included | / |
|  | VL Hendricks-Ferguson et al. | Novice Nurses' Experiences With Palliative and End-of-Life Communication. | 2015 | Journal of Pediatric Oncology Nursing | Excluded | Concept of interest |
|  | SM Herrle, B. Robinson | Helping Staff Cope with Grief | 1987 | Nursing Management | Excluded | Methodology |
|  | PS Hinds et al. | End-of-life care for children and adolescents. | 2005 | Seminars in Oncology Nursing | Excluded | Concept of interest |
|  | PS Hinds et al. | End-of-life research as a priority for pediatric oncology. | 2004 | Journal of Pediatric Oncology Nursing | Excluded | Concept of interest |
|  | PS Hinds et al. | The impact of a grief workshop for pediatric oncology nurses on their grief and perceived stress. | 1994 | Journal of Pediatric Nursing | Included | / |
|  | JM Hittle | Grieving together. | 1995 | The American Journal of Nursing | Excluded | Methodology |
|  | JB Hopkinson et al. | Everyday death: How do nurses cope with caring for dying people in hospital? | 2005 | International Journal of Nursing Studies | Excluded | Context |
|  | RN Dereen Houck | Helping nurses cope with grief and compassion fatigue: An educational intervention. | 2014 | Clinical Journal of Oncology Nursing | Excluded | Context |
|  | S. Irvin | The experiences of the registered nurse caring for the person dying of cancer in a nursing home. | 2000 | Collegian | Excluded | Concept of interest |
|  | VA Jackson et al. | A qualitative study of oncologists' approaches to end-of-life care. | 2008 | Journal of Palliative Medicine | Excluded | Concept of Interest |
|  | VA Jackson et al. | It was haunting ...: Physicians' Descriptions of Emotionally Powerful Patient Deaths. | 2005 | Academic Medicine | Excluded | Context |
|  | J. Jensen, C. Weng, HL Spraker-PerlmanHH | A Provider-Based Survey To Assess Bereavement Care Knowledge, Attitudes, and Practices in Pediatric Oncologists. | 2017 | Journal of Palliative Medicine | Excluded | Population |
|  | D. Jonas et al. | Bereavement After a Child's Death. | 2018 | Child and adolescent psychiatric clinics of North America | Excluded | Concept of interest |
|  | A. Jones | A heavy and blessed experience': a psychoanalytic study of community Macmillan nurses and their roles in serious illness and palliative care. | 1999 | Journal of Advanced Nursing | Excluded | Concept of interest |
|  | A. Jones | Group-format clinical supervision for hospice nurses. | 2006 | European Journal of Cancer Care | Excluded | Concept of interest |
|  | K. Jors et al. | Tidying rooms and tending hearts: An explorative, mixed-methods study of hospital cleaning staff's experiences with seriously ill and dying patients. | 2017 | Palliative Medicine | Excluded | Population |
|  | E. Kacel, X. Gao, HG Prigerson | Understanding bereavement: what every oncology practitioner should know. | 2011 | The Journal of Supportive Oncology | Excluded | Population |
|  | VJ Kain | An exploration of the grief experiences of neonatal nurses: A focus group study | 2013 | Journal of Neonatal Nursing | Excluded | Context |
|  | S. Kamisli et al. | Cancer patients and oncology nursing: Perspectives of oncology nurses in Turkey. | 2017 | Nigerian Journal of Clinical Practice | Excluded | Concept of interest |
|  | R. Kannai, AR Alon | Mourning My Patient, Mr Schwartz. | 2021 | Annals of Family Medicine | Excluded | Methodology |
|  | LJ Kaplan | Toward a model of caregiver grief: nurses' experiences of treating dying children | 2000 | Omega: Journal of Death and Dying | Included | / |
|  | E. Karadag et al. | Attitudes of Nurses in Turkey Toward Care of Dying Individual and the Associated Religious and Cultural Factors. | 2019 | Journal of Religion and Health | Excluded | Event |
|  | E. Kasket | Death and the Doctor II: A Phenomenological Investigation. | 2006 | Existential Analysis: Journal of the Society for Existential Analysis | Excluded | Methodology |
|  | A. Kaur, MP Sharma, SK Chaturvedi | Felt Needs of Cancer Palliative Care Professionals Working in India: A Qualitative Study. | 2021 | Indian Journal of Palliative Care | Included | / |
|  | EC Kaye | Pieces of grief | 2015 | Journal of Clinical Oncology | Excluded | Methodology |
|  | MK Kearney et al. | Self-care of physicians caring for patients at the end of life: “Being connected... a key to my survival”. | 2009 | JAMA | Excluded | Methodology |
|  | EA Keene et al. | Bereavement debriefing sessions: an intervention to support health care professionals in managing their grief after the death of a patient. | 2010 | Pediatric Nursing | Included | / |
|  | S. Klagsbrun | Forty-two years of death and dying: lessons learned. | 2010 | American Journal of Psychotherapy | Excluded | Methodology |
|  | S. Kochendörfer | Parents and oncology team members are getting together after the death of a child. Report from Children's hospital Tübingen. | 2002 | Klinische Pädiatrie | Excluded | Population |
|  | O. Kowalczyk et al. | Religion and Spirituality in Oncology: An Exploratory Study of the Communication Experiences of Clinicians in Poland. | 2022 | Journal of Religion and Health | Excluded | Event |
|  | W. Ko, N. Kiser-Larson | Stress Levels of Nurses in Oncology Outpatient Units. | 2016 | Clinical Journal of Oncology Nursing | Excluded | Concept of interest |
|  | BL Kpassagou, KMA Soedje | Health practitioners' emotional reactions to caring for hospitalized children in Lomé, Togo: a qualitative study. | 2017 | BMC Health Services Research | Excluded | Concept of interest |
|  | AS Kusano, T. Kenworthy-Heinige, CR Jr Thomas | Survey of bereavement practices of cancer care and palliative care physicians in the Pacific Northwest United States. | 2012 | Journal of Oncology Practice | Excluded | Population |
|  | T. Kushnir, S. Rabin, S. Azulai | A descriptive study of stress management in a group of pediatric oncology nurses. | 1997 | Cancer Nursing | Excluded | Concept of interest |
|  | JS Kutner, KM Kilbourn | Bereavement: addressing challenges faced by advanced cancer patients, their caregivers, and their physicians. | 2009 | Primary Care | Excluded | Methodology |
|  | RM Lally | Oncology nurses share their experiences with bereavement and self-care. | 2005 | ONS News | Excluded | Methodology |
|  | M. Lange, B. Thom, NE Kline | Assessing nurses' attitudes toward death and caring for dying patients in a comprehensive cancer center. | 2008 | Oncology Nursing Forum | Excluded | Event |
|  | R. Laor-Maayany et al. | Compassion fatigue among oncologists: the role of grief, sense of failure, and exposure to suffering and death. | 2020 | Supportive Care in Cancer | Included | / |
|  | P. Laporte et al. | Nurses: The suffering and challenges of facing death on a daily basis. | 2015 | Les infirmieres et la mort au quotidien: Souffrances et enjeux | Excluded | Methodology |
|  | D. Lathrop | Disenfranchised Grief and Physician Burnout | 2017 | The Annals of Family Medicine | Excluded | Methodology |
|  | M. Lee et al. | How Do Oncology Nurses Cope With the Psychological Burden of Caring for Dying Patients? | 2023 | Cancer Nursing | Included | / |
|  | JP Lemkau et al. | A questionnaire survey of family practice physicians’ perceptions of bereavement care | 2000 | Archives of Family Medicine | Excluded | Context |
|  | SB Lenart et al. | Grief support for nursing staff in the ICU | 1998 | Journal for Nurses in Professional Development | Excluded | Context |
|  | LE Learea, BF LiMauro | Grief among healthcare workers: a comparative study. | 1982 | Journal of Gerontology | Excluded | Context |
|  | AE Lewis | Reducing burnout: development of an oncology staff bereavement program. | 1997 | Oncology Nursing Forum | Excluded | Concept of interest |
|  | D. Lindberg | When your patients die: living with cumulative grief. | 2012 | ONS Connect | Excluded | Methodology |
|  | E. Lindemann | Symptomatology and management of acute grief | 1944 | American Journal of Psychiatry | Excluded | Concept of interest |
|  | KP Loh et al. | Associations of caregiver-oncologist discordance in prognostic understanding with caregiver-reported therapeutic alliance and anxiety. | 2020 | Journal of Pain and Symptom Management | Excluded | Concept of interest |
|  | CG Loiselle, MM Sterling | Views on death and dying among health care workers in an Indian cancer care hospice: balancing individual and collective perspectives. | 2012 | Palliative Medicine | Excluded | Population |
|  | L. Lyckholm | Dealing with stress, burnout, and grief in the practice of oncology. | 2001 | The Lancet Oncology | Excluded | Methodology |
|  | PK Maciejewski et al. | An empirical examination of the stage theory of grief. | 2007 | JAMA | Excluded | Event |
|  | CF Macpherson | Peer-supported storytelling for grieving pediatric oncology nurses. | 2008 | Journal of Pediatric Oncology Nursing | Included | / |
|  | N. Manos, J. Christakis | Coping with cancer: psychological dimensions. | 1985 | Acta Psychiatrica Scandinavica | Excluded | Concept of interest |
|  | KB Martin, CM Berchulc | The effect of dying and death on therapists. | 1988 | Physical & Occupational Therapy in Geriatrics | Excluded | Methodology |
|  | P. McGrath | Dying in the curative system: the haematology/oncology dilemma. Part 2. | 2002 | The Australian Journal of Holistic Nursing | Excluded | Concept of interest |
|  | CM McNeil | What Have We Got to Lose? | 2016 | Journal of Clinical Oncology | Excluded | Methodology |
|  | A. Medisauskaite et al. | Reducing burnout and anxiety among doctors: Randomized controlled trial. | 2019 | Psychiatry Research | Excluded | Context |
|  | A. Medisauskaite et al. | Prevalence of oncologists in distress: Systematic review and meta-analysis. | 2017 | Psycho-Oncology | Excluded | Concept of interest |
|  | SE Merel et al. | Providers' beliefs about expressing condolences to the family of a deceased patient: A qualitative and quantitative analysis. | 2015 | Journal of Palliative Medicine | Excluded | Concept of interest |
|  | J. Mirlashari, F. Warnock, J. Jahanbani | The experiences of undergraduate nursing students and self-reflective accounts of first clinical rotation in pediatric oncology. | 2017 | Nurse Education in Practice | Excluded | Population |
|  | K. Moody et al. | Pediatric palliative care. | 2011 | Primary Care | Excluded | Concept of interest |
|  | P. Moorehead | Princess Abra. | 2008 | Annals of Internal Medicine | Excluded | Methodology |
|  | B. Moreno-Milan et al. | Meaning of work and personal protective factors among palliative care professionals. | 2019 | Palliative & Supportive care | Excluded | Concept of interest |
|  | SE Morris et al. | Remembrance: A Self-Care Tool for Clinicians. | 2019 | Journal of Palliative Medicine | Excluded | Methodology |
|  | BM Mount | Dealing with our losses. | 1986 | Journal of Clinical Oncology | Excluded | Methodology |
|  | AC Muriel et al. | The "liaison" in consultation-liaison psychiatry: Helping medical staff cope with pediatric death. | 2018 | Child and Adolescent Psychiatric Clinics of North America | Excluded | Methodology |
|  | M. Murphy | A model to help nurses caring for patients who are terminally ill. | 2003 | Professional Nurse | Excluded | Concept of interest |
|  | B. Muskat et al. | The experiences of physicians, nurses, and social workers providing end-of-life care in a pediatric acute-care hospital. | 2020 | Death Studies | Excluded | Context |
|  | EM Mutto et al. | Nursing education: The experience, attitudes, and impact of caring for dying patients by undergraduate Argentinian nursing students. | 2010 | Journal of Palliative Medicine | Excluded | Population |
|  | D. Nathoo, J. Ellis | Theories of loss and grief experienced by the patient,family, and healthcare professional: a personal account of a critical event | 2019 | Journal of Cancer Education | Excluded | Methodology |
|  | RA Neimeyer | Searching for the meaning of meaning: Grief therapy and the process of reconstruction | 2000 | Death Studies | Excluded | Event |
|  | LXL Ngiam et al. | Impact of caring for terminally ill children on physicians: A systematic scoping review. | 2021 | American Journal of Hospice & Palliative Medicine | Excluded | Event |
|  | F. Oflaz et al. | A survey of emotional difficulties of nurses who care for oncology patients. | 2010 | Psychological Reports | Excluded | Concept of interest |
|  | H. Otani et al. | The death of patients with terminal cancer: the distress experienced by their children and medical professionals who provide the children with support care. | 2019 | BMJ Supportive & Palliative Care | Excluded | Event |
|  | S. Öcalan et al. | First death experiences of newly graduated nurses: A qualitative phenomenological study. | 2023 | Death Studies | Excluded | Context |
|  | CL Pace | Josh's time. | 2001 | Journal of Christian Nursing | Excluded | Methodology |
|  | D. Papadatou et al. | Greek nurse and physician grief as a result of caring for children dying of cancer. | 2002 | Pediatric Nursing | Excluded | Concept of interest |
|  | D. Papadatou, IM Martinson, PM Chung | Caring for dying children: a comparative study of nurses' experiences in Greece and Hong Kong. | 2001 | Cancer Nursing | Excluded | Concept of interest |
|  | D. Papadatou | A proposed model of health professionals grieving process. | 2000 | Omega: Journal of Death and Dying | Excluded | Methodology |
|  | RO Pasnau, FI Fawzy, N. Fawzy | Role of the physician in bereavement. | 1987 | The Psychiatric Clinics of North America | Excluded | Concept of Interest |
|  | N. Pattison, J. Droney, P. Gruber | Burnout: Caring for critically ill and end-of-life patients with cancer. | 2020 | Nursing in Critical Care | Excluded | Concept of interest |
|  | S. Pehlivan et al. | Relationship between death anxiety of Turkish nurses and their attitudes toward the dying patients. | 2020 | Omega: Journal of Death and Dying | Excluded | Context |
|  | RT Penson et al. | When does the responsibility of our care end: bereavement. | 2002 | The Oncologist | Excluded | Methodology |
|  | CS Phillips et al. | Riding the roller coaster: A qualitative study of oncology nurses' emotional experience in caring for patients and their families. | 2020 | Cancer Nursing | Excluded | Concept of interest |
|  | C. Phillips, B. Welcer | Songs for the Soul: A Program to Address a Nurse's Grief. | 2017 | Clinical Journal of Oncology Nursing | Excluded | Methodology |
|  | J. Plante, C. Cyr | Health care professionals' grief after the death of a child. | 2011 | Paediatrics & Child Health | Included | / |
|  | R. Pöyhia et al. | Palliative Care Volunteers Have High Workload but No Burnout: A Questionnaire Survey from Tanzania. | 2019 | Journal of Palliative Medicine | Excluded | Concept of interest |
|  | R. Powazki et al. | The care of the actively dying in an academic medical center: a survey of registered nurses' professional capability and comfort. | 2014 | The American Journal of Hospice & Palliative Care | Excluded | Concept of interest |
|  | D. Puente-Fernandez et al. | Nursing professionals' attitudes, strategies, and care practices towards death: A systematic review of qualitative studies. | 2020 | Journal of Nursing Scholarship | Included | / |
|  | TA Rando | Treatment of Complicated Mourning | 1993 | *book* | Excluded | Event |
|  | J. Rashotte, F. Fothergill-Bourbonnais, M. Chamberlain | Pediatric intensive care nurses and their grief experiences: A phenomenological study | 1997 | Heart & Lung | Excluded | Context |
|  | EM Redinbaugh et al. | Health care professionals' grief: a model based on occupational style and coping. | 2001 | Psycho-Oncology | Excluded | Methodology |
|  | EM Redinbaugh et al. | Doctors' emotional reactions to recent death of a patient: cross sectional study of hospital doctors. | 2003 | BMJ | Excluded | Context |
|  | KV Regan | Claire's garden. | 2003 | Nursing | Excluded | Methodology |
|  | FC Reid | Lived experiences of adult community nurses delivering palliative care to children and young people in rural areas. | 2013 | International Journal of Palliative Nursing | Excluded | Context |
|  | AE Rettig et al. | Describing Remembrance & Renewal: A Holistic Self-Care Program | 2023 | Journal of Holistic Nursing | Included | / |
|  | AE Rettig et al. | Remembrance and Renewal: Health Care Staff Spiritual Self-Care. | 2020 | Journal of Holistic Nursing | Included | / |
|  | C. Rikard-Bell | The impact of critical incidents in paediatric hospitals: a review. | 1994 | The Australian Journal of Advanced Nursing | Excluded | Event |
|  | M. Rittman et al. | Phenomenological study of nurses caring for dying patients. | 1997 | Cancer Nursing | Excluded | Methodology |
|  | RA Rodenbach et al. | Relationships between personal attitudes about death and communication with terminally ill patients: How oncology clinicians grapple with mortality. | 2016 | Patient Education and Counseling | Excluded | Context |
|  | AB Rodrigues et al. | Stressing factors and coping strategies used by oncology nurses. | 2008 | Revista Latino-Americana de Enfermagem | Excluded | Concept of interest |
|  | P. Rousseau | I'm glad I have cancer. | 2010 | Palliative & Supportive Care | Excluded | Methodology |
|  | MD Ruiz-Fernandez et al. | Social acceptance of death and its implication for end-of-life care. | 2021 | Journal of Advanced Nursing | Excluded | Concept of interest |
|  | AR Saifan et al. | Exploring the psychological status of Jordanian nurses working with cancer patients. | 2019 | Journal of Nursing Management | Excluded | Concept of interest |
|  | S. Sanchez-Reilly et al. | Caring for oneself to care for others: physicians and their self-care. | 2013 | The Journal of Supportive Oncology | Excluded | Concept of interest |
|  | C. Sanderson et al. | Signs of post-traumatic stress disorder in caregivers following an expected death: a qualitative study. | 2013 | Palliative Medicine | Excluded | Concept of interest |
|  | HK Sanoff | Managing Grief, Loss, and Connection in Oncology-What COVID-19 Has Taken. | 2020 | JAMA Oncology | Excluded | Methodology |
|  | RA Sansone et al. | Physician grief with patient death. | 2012 | Innovations in Clinical Neuroscience | Excluded | Methodology |
|  | JM Saunders & SM Valente | Nurses' grief. | 1994 | Cancer Nursing | Excluded | Methodology |
|  | KJ Sawin et al. | Oncology nurse managers' perceptions of palliative care and end-of-life communication. | 2019 | Journal of Pediatric Oncology Nursing | Excluded | Population |
|  | L. Scherrer | Tod: Auswirkungen eines Tabus auf die Arbeits- und Lebensqualitaet im Pflegedienst. | 2015 | Trauma. Zeitschrift fuer Psychotraumatologie und ihre Anwendungen | Excluded | Context |
|  | AH Schmale | The dying patient. | 1980 | Advances in Psychosomatic Medicine | Excluded | Methodology |
|  | J. Schneider | Stress, Loss, and Grief | 1984 | *Book* | Excluded | Concept of interest |
|  | R. Schulz, D. Aderman | How the medical staff copes with dying patients: a critical review. | 1976 | Omega: Journal of Death and Dying | Excluded | Methodology |
|  | R. Sedhom, A. Gupta, J. Von Roenn | Case for Focused Bereavement Education in Oncology Training. | 2021 | Journal of Clinical Oncology | Excluded | Population |
|  | JGM de Sena et al. | The care for oncologic patients undergoing pediatric palliative care and the griefs of a health team. | 2023 | Psicooncología | Included | / |
|  | JR Serwint et al. | "I learned that no death is routine": description of a death and bereavement seminar for pediatrics residents | 2002 | Academic Medicine | Excluded | Methodology |
|  | A. Shai, G. Hirschberger | On death and fear: a personal reflection on the value of social psychology research to the practice of oncologists. | 2013 | Journal of Clinical Oncology | Excluded | Methodology |
|  | T. Shanafelt, A. Adjei, FL Meyskens | When your favorite patient relapses: physician grief and well-being in the practice of oncology | 2003 | Journal of Clinical Oncology | Excluded | Methodology |
|  | M. Shayne, TE Quill | Oncologists responding to grief. | 2012 | Archives of Internal Medicine | Excluded | Methodology |
|  | H. Shi et al. | Grief as a mediator of the relationship between empathy and compassion fatigue. | 2022 | Psycho-Oncology | Included | / |
|  | K. Shimoinaba et al. | Staff grief and support systems for Japanese health care professionals working in palliative care | 2009 | Palliative & Supportive Care | Excluded | Context |
|  | AG Shuman | Learning from gus. | 2014 | The Oncologist | Excluded | Methodology |
|  | EC Small et al. | Ovarian carcinoma: management of stress in patients and physicians. | 1983 | Gynecologic Oncology | Excluded | Concept of interest |
|  | H. Smith | Childhood cancer and the role of the school nurse: Part 2. Coping with death and dying. | 1988 | Health Visitor | Excluded | Concept of interest |
|  | R. Spencer | A piece of my mind. Portrait of an artist. | 2013 | JAMA | Excluded | Methodology |
|  | L. Spencer | How do nurses deal with their own grief when a patient dies on an intensive care unit, and what help can be given to enable them to overcome their grief effectively? | 1994 | Journal of Advanced Nursing | Excluded | Context |
|  | R. Srivastava | The Sharing of Loss. | 2016 | The New England Journal of Medicine | Excluded | Methodology |
|  | J. Stein et al. | The bereavement visit in pediatric oncology | 2006 | Journal of Clinical Oncology | Excluded | Methodology |
|  | TM Stephany | Michael's sign. | 1993 | The American Journal of Nursing | Excluded | Methodology |
|  | YN Stokar et al. | Themes of end-of-life care in memorable cases of medical health professionals: A mixed methods approach. | 2022 | Current Psychology | Excluded | Event |
|  | SJ Stowers | Nurses cry too, being exposed to death and loss - How do we deal with our own grief? | 1983 | Nursing Management | Excluded | Methodology |
|  | O. Taubman-Ben-Ari et al. | Meaning in life and personal growth among pediatric physicians and nurses. | 2008 | Death Journals | Excluded | Concept of interest |
|  | RJ Topf, E. Bergstraesser | Palliative Betreuung und Behandlung. (chapter) | 2014 | Das krebskranke Kind und sein Umfeld. Psychosoziale Aspekte der Versorgung und Unterstützung. | Excluded | Concept of interest |
|  | S. Tranter, E. Josland, K. Turner | Nurses' bereavement needs and attitudes towards patient death: A qualitative descriptive study of nurses in a dialysis unit | 2016 | Journal of Renal Care | Excluded | Context |
|  | K. Treggalles et al. | An exploration of the lived experience of professional grief among occupational therapists working in palliative care settings. | 2018 | Australian Occupational Therapy Journal | Included | / |
|  | JW Tsai | Processing Death. | 2017 | JAMA Oncology | Excluded | Methodology |
|  | PR Tutelman et al. | It could have been me: An interpretive phenomenological analysis of health care providers' experiences caring for Adolescents and Young Adults with terminal cancer. | 2019 | Journal of Adolescent and Young Adult Oncology | Excluded | Event |
|  | CC Tye | Sudden bereavement in accident and emergency: The development and evaluation of a short educational course for qualified nurses | 1996 | Accident and Emergency Nursing | Excluded | Context |
|  | PA Tyler, RN Ellison | Sources of stress and psychological well-being in high-dependency nursing. | 1994 | Journal of Advanced Nursing | Excluded | Concept of interest |
|  | ML Vachon | Staff stress in hospice/palliative care: a review. | 1995 | Palliative Medicine | Excluded | Event |
|  | IA van Duin, AA Kaptein | Scared witless about death--ovarian cancer narratives compared. | 2013 | Journal of Cancer Education | Excluded | Concept of interest |
|  | RT Vogel | Todesthemen in der Psychotherapie. | 2012 | *book* | Excluded | Context |
|  | A. Wakefield | Nurses' responses to death and dying: a need for relentless self-care. | 2000 | International Journal of Palliative Nursing | Excluded | Methodology |
|  | AM Watson | Let It Be Hard. | 2015 | Journal of Clinical Oncology | Excluded | Methodology |
|  | J. Wenzel et al. | Working through grief and loss: Oncology nurses' perspectives on professional bereavement. | 2011 | Oncology Nursing Forum | Included | / |
|  | L. Wilde, B. Worster, D. Oxman | Monthly “grief rounds” to improve residents’ experience and decrease burnout in a medical intensive care unit rotation | 2016 | American Journal of Medical Quality | Excluded | Context |
|  | BM Wolpin et al. | Learning to cope: how far is too close? | 2005 | The Oncologist | Excluded | Methodology |
|  | HL Wu, DL Volker | Living with death and dying: the experience of Taiwanese hospice nurses. | 2009 | Oncology Nursing Forum | Excluded | Concept of interest |
|  | MH Yang, S. McIlfatrick | Intensive care nurses' experiences of caring for dying patients: a phenomenological study. | 2001 | International Journal of Palliative Nursing | Excluded | Concept of interest |
|  | J. Yi et al. | When does compassion fatigue hit social workers? Caring for oncology patients in Korea. | 2018 | Qualitative Social Work | Excluded | Concept of interest |
|  | SC Zambrano et al. | Attending patient funerals: Practices and attitudes of Australian medical practitioners. | 2017 | Death Studies | Excluded | Concept of interest |
|  | SC Zambrano, A. Chur-Hanson, GB Crawford | The experiences, coping mechanisms, and impact of death and dying on palliative medicine specialists | 2013 | Palliative & Supportive Care | Included | / |
|  | SC Zambrano, A. Chur-Hanson, GB Crawford | On the emotional connection of medical specialists dealing with death and dying: a qualitative study of oncologists, surgeons, intensive care specialists and palliative medicine specialists. | 2012 | BMJ Supportive & Palliative Care | Excluded | Context |
|  | B. Zhang, A. El-Jawahri, HG Prigerson | Update on bereavement research: evidence based guidelines for the diagnosis and treatment of complicated bereavement. | 2006 | Journal of Palliative Medicine | Excluded | Event |
|  | R. Zheng et al. | How new graduate nurses experience patient death: A systematic review and qualitative meta-synthesis. | 2016 | International Journal of Nursing Studies | Excluded | Context |
